# Cancer driver genes: a guilty by resemblance doctrine

**DOI:** 10.7717/peerj.6979

**Published:** 2019-06-25

**Authors:** Emilie Ramsahai, Vrijesh Tripathi, Melford John

**Affiliations:** 1 Department of Mathematics and Statistics, The University of the West Indies, St. Augustine, Trinidad and Tobago; 2 Department of Preclinical Sciences, The University of the West Indies, St. Augustine, Trinidad and Tobago

**Keywords:** Signal transduction, Network topology, m-reach, Cancer, Novel driver genes, Pathways

## Abstract

A major benefit of expansive cancer genome projects is the discovery of new targets for drug treatment and development. To date, cancer driver genes have been primarily identified by methods based on gene mutation frequency. This approach fails to identify culpable genes that are not mutated, rarely mutated, or contribute to the development of rare forms of cancer. Due to the complexity of the disease and the sheer volume of data, computational methods may encounter a NP-complete problem. We have developed a novel pathway and reach (PAR) method that employs a guilty by resemblance approach to identify cancer driver genes that avoids the above problems. Essentially PAR sifts through a list of genes of biological pathways to find those that are common to the same pathways and possess a similar 2-reach topology metric as a reference set of recognized driver genes. This approach leads to faster processing times and eliminates any dependency on gene mutation frequency. Out of the three pathways, signal transduction, immune system, and gene expression, a set of 50 candidate driver genes were identified, 30 of which were new. The top five were *HGF*, *E2F1*, *C6*, *MIF*, and *CDK2*.

## Introduction

An understanding of the genetic causes of cancer is a prerequisite for the administration of effective treatment regimes and the development of new drugs. The proliferation of normal cells is initiated and controlled by the actions of complex signaling pathways that pass messages between cells and between proteins inside cells. Cancer cells are different from normal cells in that they develop the capability to initiate their own cell division and ignore messages to stop dividing or to commit suicide via apoptosis. These defects are brought about by random genetic alterations that by chance rewire signaling pathways in a manner that favors uncontrolled cell division.

Genes that are frequently mutated in tumors are readily identified as cancer driver genes. However, this approach fails to identify oncogenes that are activated by increased expression, tumor suppressor genes (TSGs) that are deactivated by suppressed expression, or driver genes that are rarely mutated. Expression levels of genes may be altered by methylation of their promoters or by the binding of micro RNA molecules to their messenger RNA molecules (Cancer Genome Atlas Network, 2012; Cancer Genome Atlas Research Network, 2008). Using computational processing, lists of driver genes have been generated based on properties such as mutation frequency, conserved regions, protein function, mutation patterns, and protein interactions (Hofree et al., 2016; Melloni et al., 2014). The small overlap between such lists highlights the limitations of current methods; a major problem being the validation of candidate driver genes for which there are no definitive means (Tokheim et al., 2016). Benchmarking may be carried out by comparison with the Cancer Genome Census (CGC) (Futreal et al., 2004), which is curated and regularly updated. The availability of enriched datasets of cancer genome and biological pathway data provides an opportunity for the development of new methods to identify candidate cancer driver genes that are not dependent on mutation frequency.

Proteins frequently mutated in cancer have been found to have higher degrees of connectivity with other members of protein-protein interaction networks (Cui et al., 2007). Such cancer-driver proteins are often found at central locations as determined by the number of shortest paths passing through them, and are said to have high betweenness centrality (Li et al., 2013; Xia et al., 2011). Driver proteins have been found to have high clustering coefficients, which means their neighbors are also neighbors of each other (Cui et al., 2007; Li et al., 2009). Analysis of such interactions has been used to identify candidate cancer-related genes (Creixell et al., 2015; Özgür et al., 2008). Jonsson & Bates (2006) investigated the network positions of 346 driver-genes of the CGC (Futreal et al., 2004), and found that they had on average twice as many interacting partners as non-cancer genes. They found driver-genes tended to reside in larger network clusters, and to be members of more groups than other genes. By selecting proteins that had interactions with others already known to cause a disease that fell within one of the significant loci, Oti et al. (2006) were able to achieve a 10-fold enrichment of disease-causing genes compared to a random selection at the same locus. A “guilt-by-association” approach to identify lists of novel genes associated with Crohn’s disease and type 2 diabetes has been employed (Lee et al., 2011).

In this paper, we report on a novel method pathway and reach method (PAR) that assesses pathways of interest to identify those genes that are similar to a set of gold standard cancer driver genes using a guilty by resemblance doctrine.

## Materials and Methods

### Data sources

Lists of driver genes were obtained from Vogelstein et al. (2013), the CGC (Futreal et al., 2004), and Lawrence et al. (2014). The 71 genes common to all three lists constituted the dr3Genes set used as reference genes. A list of pathways and their constituent genes was obtained from the Reactome annotation package (Ligtenberg, 2017). All the Reactome pathways were used in determining the pathways a gene was a member of. Additionally, the Signal Transduction pathway, the Immune System pathway and Gene Expression pathway were analyzed in turn for novel candidate driver genes when each of these pathways were assigned as candidate genes and referred to as the cdGenes set. Three gene interaction networks were employed for the topology similarity analysis including STRING version 10.0 (Szklarczyk et al., 2014, 2017), BioGrid version 3.4.163 (Chatr-Aryamontri et al., 2017; Stark et al., 2006) and pathway commons repository (Cerami et al., 2011; Fang et al., 2016). Genes of the Reactome annotation package were mapped to genes of the interaction networks using common key values obtained from the org.Hs.eg.db package (Carlson et al., 2013) of Bioconductor (Gentleman et al., 2004).

(1) $${\rm{JSI}} = {{\left| {{\rm{Lr}}\mathop \cap {\rm{Lc}}} \right|} \over {\left| {{\rm{Lr}}\mathop \cup {\rm{Lc}}} \right|}} = {{\left| {{\rm{Lr}}\mathop \cap {\rm{Lc}}} \right|} \over {\left| {{\rm{Lr}}} \right| + \left| {{\rm{Lc}}} \right| - \left| {{\rm{Lr}}\mathop \cap {\rm{Lc}}} \right|}}$$

### Pathway similarity matrix

**Figure 1:**
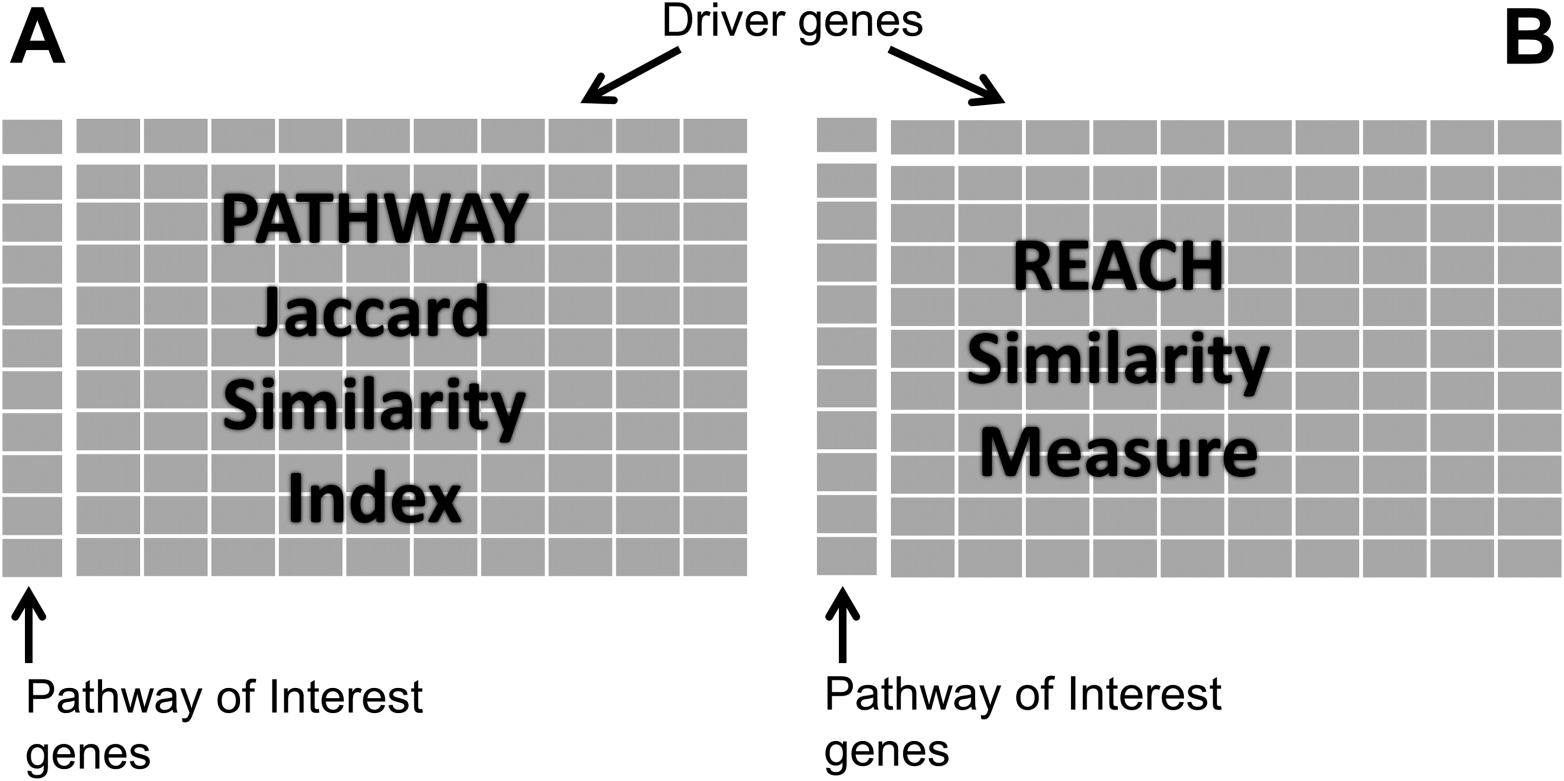
Similarity matrices. (A) Represents matrix P of pathway similarity between each gene of interest gene and every other driver gene. (B) represents matrix R of reach similarity based on equal levels between each gene of interest and every other driver gene.

Genes of the dr3Genes set were used as columns of the pathway similarity matrix, while genes of cdGenes set were used as rows. Cells of the matrix were populated with one or zero based on a jaccard similarity index (JSI) as shown in Fig. 1A. The JSI was calculated using Eq. (1) where Lr represents a list of pathways a row gene were members of, and Lc a list of pathways a column gene were members of. JSI values fall within a range of 0 to 1, where 0 indicates no similarity between the two lists and 1 indicates they are identical. By using the JSI, PAR has the flexibility to adjust the extent to which the two pathway lists must overlap before declaring them sufficiently similar. By choosing a JSI cut-off value, any values greater than or equal the cut-off indicates sufficient similarity between the two lists of pathways. In the pathway similarity matrix these were assigned a value of 1, while the others with JSI values less than the JSI cut-off were populated with a value of 0. Reactome identified a small number of overlapping pathways for the genes in the pathway similarity matrix. Because these genes belonged to a number of different pathways, 90% of the JSI values ranged from [0.0036, 0.1533] for pathways which overlapped. Therefore, this implementation of PAR, required a JSI cut-off value of 0.0036 (indicative of the existence of at least one common pathway). The matrix values were assigned 1 if a row gene belonged to one or more pathways as a column gene.

Depending on the pathway listings being used the JSI cut-off can be adjusted. Large-scale pathway reaction and interaction data continue to be produced using different network modeling techniques applied on experimental data (Kumar & Ranganathan, 2013). Additionally, a number of statistical methods have been proposed to estimate gene interaction networks from expression and other high-dimensional heterogeneous data (Lee et al., 2017; Yu et al., 2015). This coupled with the on-going refinement and discovery of new pathways, can lead to PAR using a higher JSI cut-off value.

### Reach similarity matrix

The number of genes reachable from each gene two steps out was counted, and divided by the total number of genes in the network to calculate 2-reach values for each node in the gene interaction network. This metric is used to rank genes based on the proportion of the network accessible in two steps. A node with a 2-reach value of 1, has access to the whole network in two steps, whereas a 2-reach value of 0.5 indicates only half the network is accessible from that node in two steps. All the nodes in the network can be sorted based on their 2-reach values. This sorted list of nodes when divided into four equal segments, each containing the same number of nodes gives rise to four levels. Those with the highest reach values are at Level 1, the next segment would be Level 2, followed by Level 3 and those with the lowest reach values are at Level 4. Level 1 was thus assigned to genes of high connectivity, while Level 4 was assigned to genes of low connectivity.

Genes of the dr3Genes set were used as columns of the reach similarity matrix, while genes of the cdGenes set were used as rows. Cells of the matrix were populated with 0 or 1 values based on comparisons of reach values. Cells of the reach similarity matrix were populated with a value of 1 for cdGenes and dr3Genes of the same level; otherwise, they were populated with a value of 0. To compare the suitability of 1-reach and 3-reach to the 2-reach metric used, the process was repeated for 1-reach and 3-reach values, where 1-reach and 3-reach were the number of genes reachable one and three steps out from a gene, respectively. The 1-reach parameter is commonly known as the degree of the node.

### Contingency table construction

**Figure 2:**
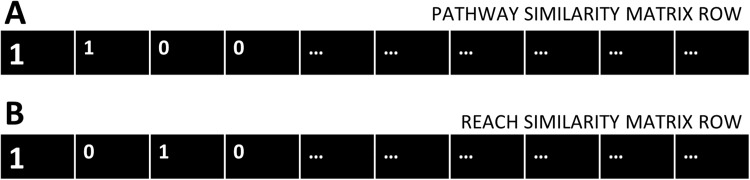
Pathway and Reach similarity rows. Each gene in the pathway of interest is associated with two rows of ones and zeros. (A) One row from the Pathway similarity Matrix P and (B) the other from the Reach similarity matrix R.

Two-by-two contingency tables were constructed for each gene of the cdGenes set, using the categorical data in the PAR similarity matrices. Each cdGene occurs both in the PAR similarity matrix as a row, Fig. 2A represents a row from the Pathway similarity matrix and Fig. 2B is from the Reach similarity matrix. These two rows are a series of zeros and ones. Across the two rows, there are a number of drGenes with a one in both the PAR matrix, as highlighted in the first column of the two rows of Fig. 2.

A count of the number of drGenes with this combination of ones in both rows, is totalled as A. Aggregate values A, B, C, and D are derived by counting matching combinations across the two rows from the pathway similarity and reach similarity matrices, where:

A = count of pathway (1) and reach (1) combination (as seen in column 1)
B = count of pathway (1) and reach (0) combination (as seen in column 2)
C = count of pathway (0) and reach (1) combination (as seen in column 3)
D = count of pathway (0) and reach (0) combination (as seen in column 4)

This count of the number of occurrences of the four possible combinations of zeros and ones (11, 10, 01, and 00) in the two rows can be represented as a contingency table as shown in Table 1.

**Table 1:** Two-by-two contingency table.

Contingency table for one gene from the cdGenes set	Reach (1)	Reach (0)
Pathway (1)	A	B
Pathway (0)	C	D

### Identification of driver genes

The Fisher’s exact test takes a contingency table as its input and computes a *p*-value. This was repeated 1,000 times to produce a set of 1,000 *p*-values for each cdGene. On each repetition, we used a random subset of 50 genes from the dr3Genes set. This matrix of *p*-values, consisted of the cdGenes as rows and 1,000 columns of *p*-values. Lastly, these resulting nominal *p*-values were adjusted to control the false discovery rate using the Benjamini–Hochberg procedure for multiple-testing correction.

**Figure 3:**
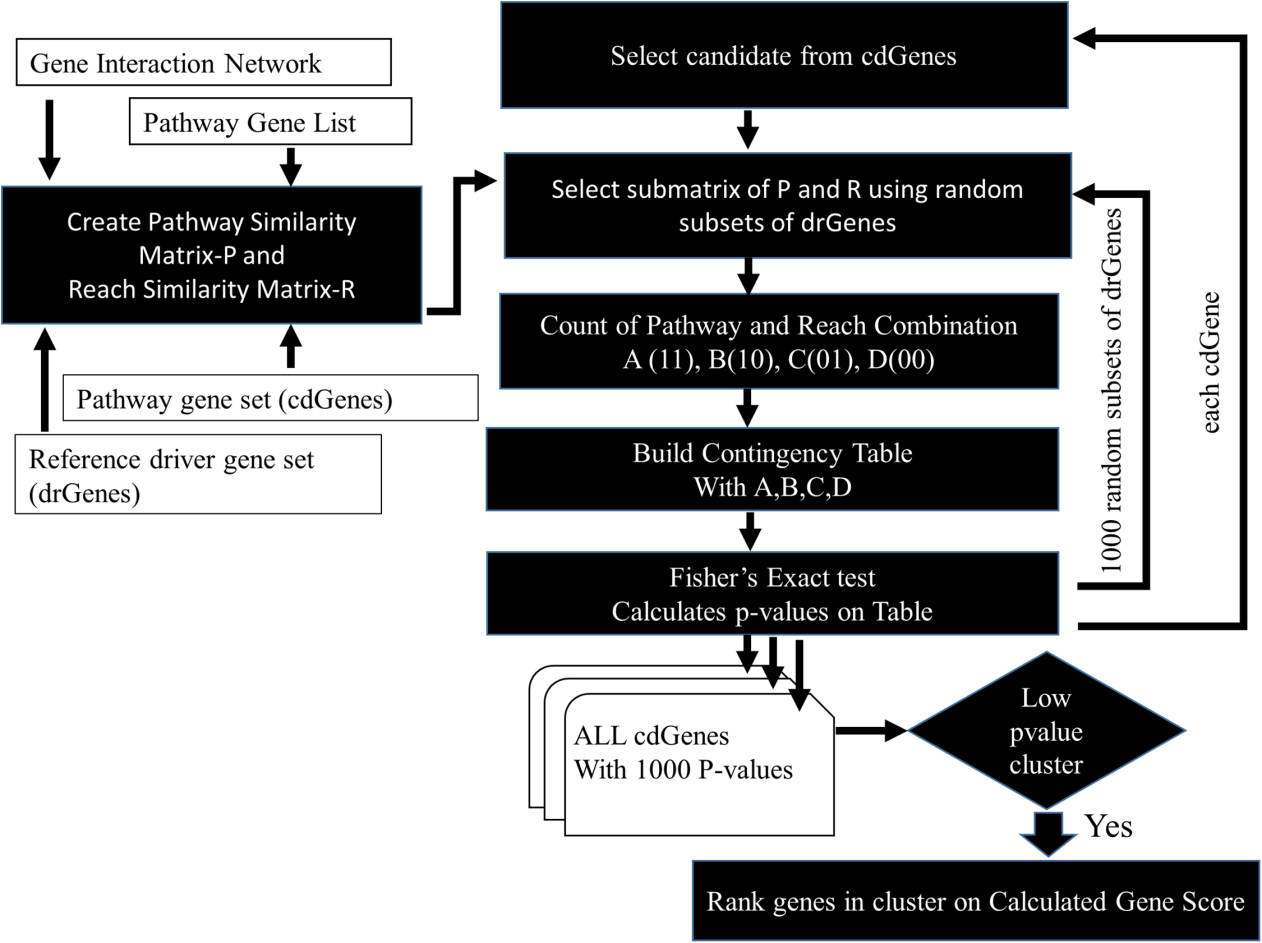
PAR flowchart. PAR driver gene prediction method pseudocode flowchart.

The similarity between cdGenes and dr3Genes concerning pathway membership and gene network 2-reach values was assessed so those that were similar could be assigned driver gene status by PAR see Fig. 3. For this purpose, pathway similarity and reach similarity matrices consisting of all the cdGenes of a pathway as rows and a random set of dr3Genes as columns were combined using a contingency table. These contingency tables were the input to the Fisher’s exact test and *p*-values were calculated. This process was repeated 1,000 times with different subsets of well-known driver genes to obtain 1,000 *p*-values. By determining which genes were the most similar to well-known cancer annotated genes, using the PAR metric we hypothesized these genes are also cancer drivers. PAR pseudocode block diagram is presented in Fig. 3, which was implemented using R. File S3 includes R code and inline results.

### Ranking genes

The corrected *p*-value matrix was used to produce a heatmap. Individual values contained in this matrix are represented as colors. The row of the heatmap is labeled with the cdGenes while each column represents one set of the 1,000 *p*-values. Individual *p*-values are assigned different colors across a spectrum, from red representing a *p*-value of zero and green a *p*-value of one. The heatmap function also reorders the genes to ensure those genes that cluster about a low *p*-value are placed together on the heatmap forming a red band.

A *p*-value is a number between zero and one, and in standard statistical practice a small *p*-value usually less than or equal to 0.05 indicates significance. Clusters of genes with similar or vastly different *p*-values are visible from a heatmap. Each of the genes had multiple measures of similarity across the 1,000 randomly selected driver gene list. Genes that consistently had low *p*-values across the 1,000 calculations were candidates for driver gene status. A frequency distribution of the *p*-values of these genes highlights the number of *p*-values falling below 0.05, thereby representing their significance as candidate driver genes. The genes in the red cluster were ranked based on two contributing factors (1) high frequency of *p*-values less than or equal to 0.05 and (2) a low mean *p*-value across the 1,000 runs.

(2) $${\rm{Gene\ score }} = {\rm{ }}{{\left({{\rm{Count}}\ \left({p\text{-} {\rm{values}} > 0.05} \right)} \right)} \over {1,000}}{\rm{ }} + {\rm{ Mean}}\ \left({p\text{-} {\rm{values}}} \right)$$

The first factor ranking is based on a high value while the 2nd factor ranking is based on a low value. To combine these two factors, they both needed to be based on a low value. The first term (1) high frequency of *p*-values less than or equal to 0.05 was adjusted to the low frequency of *p*-values greater than 0.05. A high frequency of *p*-values less than or equal to 0.05, implies a low frequency of *p*-values greater than 0.05. This frequency can be normalized to lie in the range of [0,1] by dividing by the total number of *p*-values per gene (1,000). The gene score (GS) was calculated using Eq. (2). Lower scoring genes, were considered more significant and prioritized to be nominated as members of the first list of candidate drivers.

### Literature mining validation

Literature mining was used to determine which of the candidate driver genes identified by the PAR computational method, had recent supporting experimental evidence of its role as a driver gene. Extraction of abstracts of cancer-associated genes through automated PubMed queries and analysis of these results using text mining gave us the opportunity to look at the significance of the first list of candidate driver genes.

A PubMed database search allowed us to query on the title and abstract only using key words. The exact terms used for the initial search space were:

Cancer AND patient.

This extracted all the papers with the word “Cancer” and “Patient” in the Title or Abstract of the paper from the PubMed repository. These abstracts were saved to a text file. By implementing the pub.medR (Rani, Shah & Ramachandran, 2015) text mining library we were able to look at sentences with the specific genes highlighted by the heatmap with a low GS over a 3 year time period and further confirm their importance based on how they have been recently implicated in the literature.

## Results

### Driver gene lists

**Figure 4:**
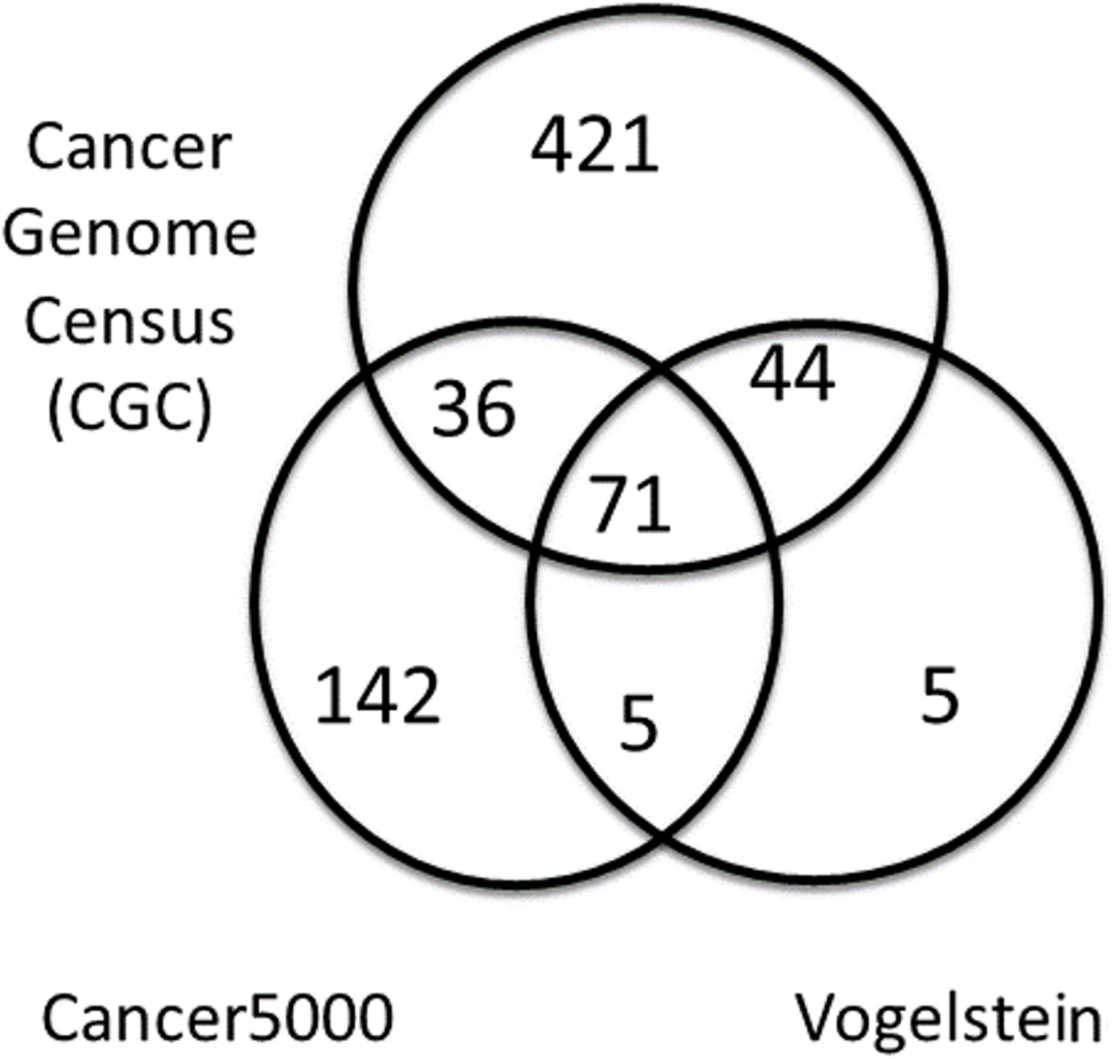
Venn diagram of well known driver gene list. Venn diagram showing overlap of three lists of driver genes.

A summary of the three lists of driver genes from Vogelstein et al. (2013), the CGC (Futreal et al., 2004), and Lawrence et al. (2014) (Cancer5000) is presented in Table 2. The curated list of 572 CGC genes was much larger than the 125 genes of Vogelstein et al. and the 254 genes of Lawrence et al., both of which were generated by computation. As shown in Fig. 4, of the full list of 724 genes from all three sets, 71 (9.8%) were common to all lists. These were used as a reference set of driver genes (dr3Genes) by PAR for similarity association comparisons. Of the remaining genes, 85 (11.7%) were common to just two of the three lists, and 568 (78.5%) were present in only one list. The 156 genes that were present in at least two lists were also used as a reference set of driver genes (dr2Genes).

**Table 2:** Published lists of driver genes.

Driver list	Origin	No. of genes	Last update	No. of citations
Vogelstein	Computational	125	2013	1,615
Cancer5000	Computational	254	2014	958
CGC-Cancer Genome Census	Multiple experiments-literature	572	2015	1,922

### Pathways and interaction networks

The number of driver genes of the dr3Genes set that were present in Reactome pathways was determined. The three pathways in Table 3 containing the highest number of known driver genes were Signal Transduction with 239, Immune System with 153, and Gene Expression with 173 across the STRING network; STRING being one of the three networks used in our analysis. There were significant differences between the three interaction networks. With a total of 2,232,450 edges, the STRING network had the largest number of interactions. The BioGrid network contained 721 well know driver genes, which was more than the other two networks (see Table 4).

**Table 3:** Pathway structure comparison of the three networks.

Reactome pathways	Signal transduction	Immune system	Gene expression
Pathway commons			
#of Nodes	2,150	1,494	1,030
#of Interactions	38,489	16,586	14,096
#known drivers	230	143	161
BioGrid			
#of Nodes	2,135	1,917	1,272
#of Interactions	49,383	36,579	34,510
#known drivers	233	151	169
STRING			
#of Nodes	2,655	1,970	1,410
#of Interactions	2,07,992	74,306	90,635
#known drivers	239	153	173

**Table 4:** Structure of the three networks.

Network	Pathway Commons	BioGrid	STRING
#of Nodes	15,044	22,061	17,397
#of Interactions	2,49,606	428,140	2,232,405
#drivers	666	721	717
Density	0.0011	0.0017	0.0147
Diameter	14	8	7

Reactome provided the list of genes associated with pathways. With a list of genes, these complete networks could be split into fragments. The main fragment being the genes and interactions for a pathway. Therefore Reactome provided the list of genes for a pathway, and a pathway subnetwork was extracted from the complete interaction networks. Within each of the three networks we observed the three subnetworks associated with the three main pathways of interest: signal transduction, immune system, and gene expression. The signal transduction pathway contained more driver genes, and was by far the largest subnetwork based on number of nodes and interactions. The Immune System pathway was larger than the Gene Expression pathway based on the number of nodes, but the latter was found to have more well known driver genes (see Table 4).

All three of the networks contained a significant number of driver genes in all of the three pathways. The structure of the STRING, BioGrid, and Pathway Commons networks were different as outlined in Table 4, but each of them contained a significant number of well known driver genes for each of the three pathways as seen in Table 3. For instance the Signal Transduction pathway extracted from the Pathway Commons network contained 230 known driver genes, while the BioGrid version of this pathway had just three more driver genes and the STRING network six more. All three networks were used in this analysis. Additionally due to the significance of the deregulation of the three pathways to the development of cancer and the high number of interactions their full compliment of genes was selected for processing and each in turn assigned to the cdGenes set for analysis by PAR to identify new candidate cancer driver genes.

### Reach topology levels

**Figure 5:**
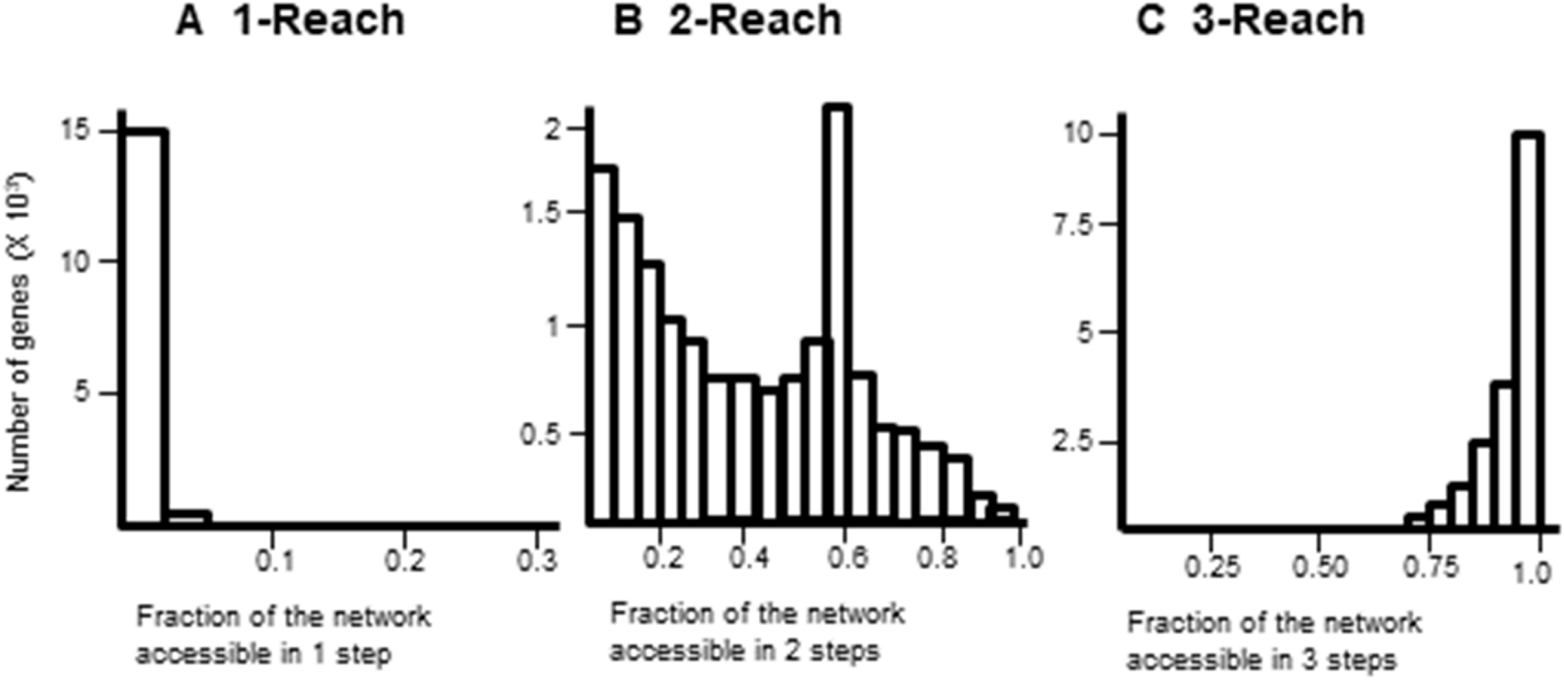
Comparison of M-Reach for *M* = 1, 2, 3. Frequency distribution of reach values of the total network for 1-reach (A), 2-reach (B), and 3-reach parameters (C).

**Figure 6:**
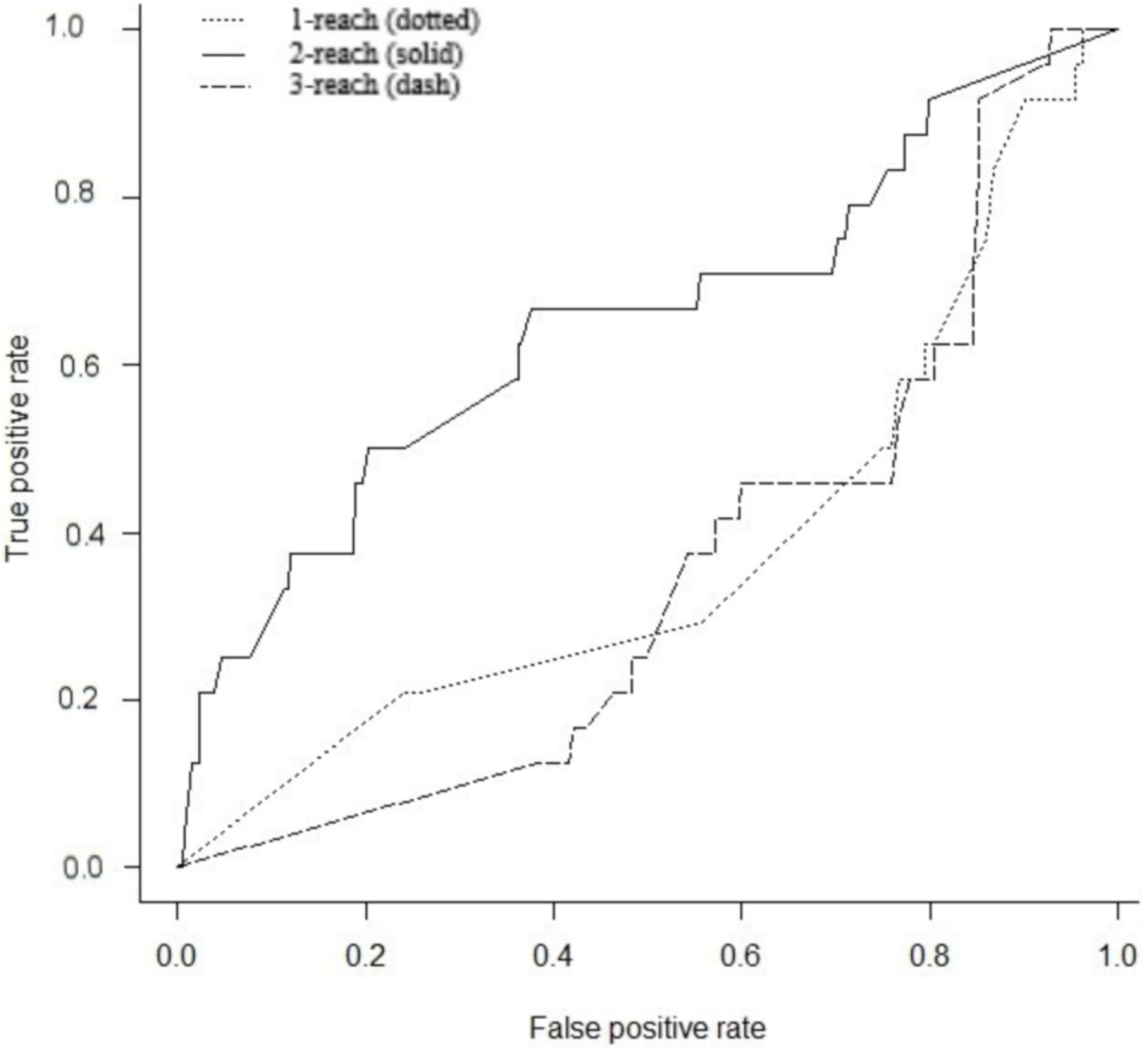
Receiver operator characteristic (ROC). Comparison of PAR for M-Reach where *M* = 1, 2, 3. ROC for PAR using 1-reach (dotted), 2-reach (solid), and 3-reach (dash) parameters.

Gene interaction networks have been observed to follow the small world phenomena where it is possible to reach every other node from a single node in a small number of steps (Newman, 2003). The percentage of nodes accessible one, two, and three steps away from a single node are termed 1-reach, 2-reach, and 3-reach, respectively. These values were calculated for each gene of the cdGenes set. The frequency distributions of 1-reach, 2-reach, and 3-reach values are presented in Figs. 5A–5C, respectively. The 1-reach and 3-reach graphs both showed non-uniform distributions that were highly skewed in contrast to the 2-reach graph, which was more evenly distributed. This broader distribution made 2-reach a more suitable parameter for PAR to distinguish between driver and non-driver genes. This was confirmed by comparing how many driver genes from the dr2Genes set PAR was able to correctly predict using each of the reach parameters in turn. Results are presented in the ROC curve of Fig. 6, which shows better predictions were obtained for 2-reach.

The three networks contained different numbers of genes, with varying reach values. Each level in the STRING network contained 4,349 genes, while the BioGrid network had 5,515 and the Pathway commons 3,761 genes in each quantile (see Table 5). The Pathway commons network reach values were more evenly distributed across the whole network, while both the BioGrid and STRING network were skewed, but in opposite directions. The BioGrid network with less than a quarter of the network with a 2-reach value greater than 0.5 in Level 1. While the STRING network had less than a quarter of the network with a low 2-reach value, those genes were in Level 4 only. Both Pathway Commons and the STRING network had nodes that attained a reach value greater than 0.98, indicating there were nodes with almost or full access to all other nodes in these networks in two steps (See Table 5).

**Table 5:** Reach cutoff levels of the three networks.

2-Reach	Pathway Commons	BioGrid	STRING
Level 1	0.983780	0.760029	0.985572
Level 2	0.511699	0.169031	0.744783
Level 3	0.368718	0.073024	0.658504
Level 4	0.128871	0.019174	0.299132
	0.000199	0.000045	0.000114
No. of nodes per level/quantile	3,761	5,515	4,349

### Heatmaps

**Figure 7:**
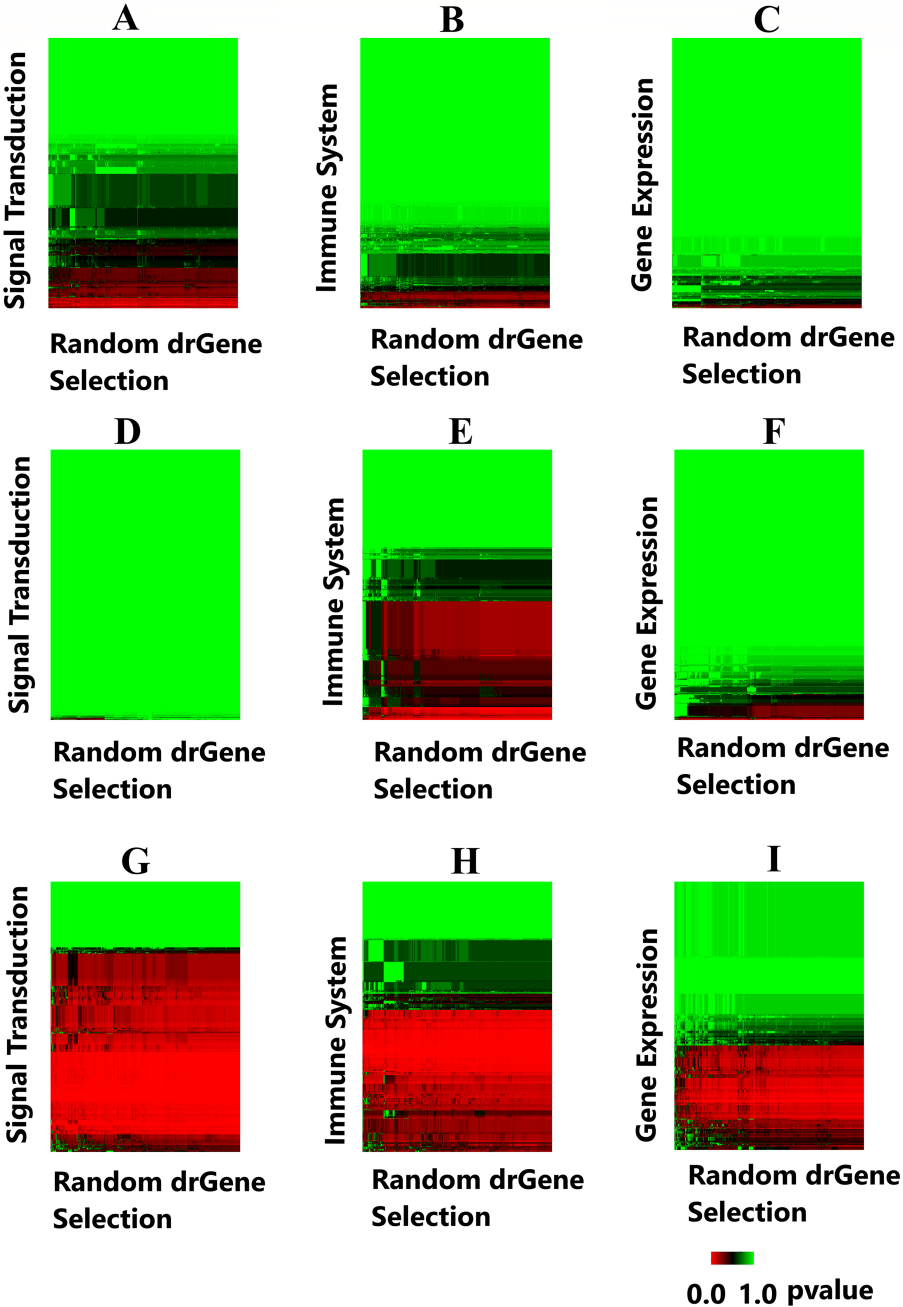
Heatmaps highlight candidate driver genes. Each of the nine heatmaps are assessed to determine candidate genes from the solid red band. (A) Signal Transduction pathway from the Pathway Commons network; (B) Immune System pathway from the Pathway Commons network; (C) Gene Expression pathway from the Pathway Commons network. (D) Signal Transduction pathway from the BioGrid network; (E) Immune System pathway from the BioGrid network; (F) Gene Expression pathway from the BioGrid network. (G) Signal Transduction pathway from the STRING network; (H) Immune System pathway from the STRING network; (I) Gene Expression pathway from the STRING network.

A heatmap was produced to highlight the clustering of the cdGenes based on their similarity to the 71 driver genes of the dr3Genes set. After the BH multiple test comparison, we see the genes that were consistently similar across the 1,000 combinations of driver gene list form a red band across the heatmap. Each of the 1,000 columns of the heatmap is associated with a random selection from the dr3Genes. Figure 7 shows the heatmaps of the three pathways (Signal Transduction, Immune System, and Gene Expression) for the three networks. Figures 7A–7C are the heatmaps for the Pathway Commons network, Figs. 7D–7F for the BioGrid and Figs. 7G–7I was generated using the STRING network. These nine heatmaps are used based on their similarity to the 1,000 randomly selected lists of well-known driver genes. The heatmap clustering algorithm is able to assess outlier *p*-values when grouping genes. It highlighted the red band as the main group of interest with few outliers. The dendrogram allowed access to the red band cluster of genes. The STRING network produced the largest red band cluster for all three of the pathways.

The genes in the red band of the nine heatmap clusters were sorted by their GS score, those with GS scores greater than one were removed. The remaining genes were preferential contenders. A number of these were themselves well known driver genes, the remaining formed the first list of candidate driver genes. This list belonged to similar pathways as known driver genes, and also demonstrated similar levels in the gene interaction network. The File S1 provides the 1,000 *p*-values and the GSs with its components. Additionally, File S1.2 provides the corresponding frequency distribution graphs for 50 genes with the lowest GSs. It is noted these distributions are all skewed toward the left, as there are more *p*-values close to zero for these genes.

### Literature validation

From the three main lists nominated for candidate driver gene status from the three pathways, derived using the heatmap and the GS, 20 were known driver genes that included well annotated driver genes like *KRAS NRAS*, *HRAS*, *FOXO1*, and *FOXO3*. A literature search revealed each of the remaining genes has been implicated in the development of cancer in at least one study.

To further systematically assess these new candidates, we used the PubMed repository. From January 2016 to August 2018, 46,400 plus papers were associated with cancer patients, the abstract of which were saved to a text file that required 128.4 MB of storage space. By implementing the pub.medR text-mining library, we were able to look at sentences, with the specific genes and further confirm their importance. The Table in File S5 presents the time series data for the candidate genes that were significantly associated with cancer patients, and as a result, appeared in the title or abstract of those research papers. File S2 provides these sentences, ordered by the year the research papers were published: 2016, 2017, and 2018, respectively. This file contains the sentences and PubMed Ids at the end of each gene sentence description. The six genes that were referenced in abstracts are shortlisted. Curiously, PAR identified the well-known oncogene SRC, which was not in any of the three published lists of driver genes used in our research. It has since been added to the CGC list, which has grown from 572 in November 2015 to 616 at the time of writing this paper. *HGF*, *E2F1*, and *C6* have been referenced in more than one abstract in the current year of 2018. While we also consider *MIF* and *CDK2* to be strong candidate driver genes due to research published in the last 18 months.

### Comparison of driver gene prediction methods

The CGC list of cancer driver genes is actively curated using published genomic experimental data that requires at least two independent studies by different groups. Inclusion is not based on computational methods, but based on supporting evidence. The CGC list of cancer driver genes is by far the largest of the three lists employed in this study. The census requires differently expressed genes to also be mutated to be classified as driver genes.

The method employed to classify cancer driver genes by Vogelstein is computational in that it is based on the frequency of mutations. It rates genes that are frequently mutated in a large population of tumors as more likely candidates for driver gene status. Vogelstein’s method further distinguishes between oncogenes and TSGs based on characteristics of mutation. The occurrence of more than 20% of the mutations at the same position of a gene, in a given population of tumors, is used as a threshold for classification as an oncogene, whereas, a more even distribution is considered as indicative of TSG status. Vogelstein’s computation does not take into account background rates as they vary greatly among different patients and regions of the genome. While a few cancer genes are mutated in a high proportion of tumors of a given type (>20%), most are mutated at intermediate frequencies (2–20%). The problem with Vogelstein’s method is that driver genes that are not frequently mutated in tumors escape detection, as well as genes that are commonly mutated in tumors that rarely occur. As PAR does not use frequency of mutation to identify cancer driver genes, it is capable of identifying candidate driver genes that methods based on frequency are likely to miss.

In the identification of cancer driver genes, Cancer5000 employs a more complex computational method involving three previously published methods MutSigCV (Lawrence et al., 2013), MutSigCL, and MutSigFN (Lawrence et al., 2014) which measure the significance of mutation burden, clustering, and functional impact, respectively. MutSigCV provides a measure of mutational frequencies relative to background rates of non-coding regions of DNA. MutSigCL provides a measure of the tendency of mutations to cluster at particular hot spots while MutSigFN evaluates the tendency of mutations to occur at positions in evolutionarily conserved regions of proteins. The significance of a gene is determined by consideration of these three values. The computational method used to construct the Cancer5000 list is very different from that used by PAR, which does not employ any of the parameters it uses, and which considers the functional impact of genes by utilizing gene interaction networks and pathways in the identification of driver genes. Table 6 highlights the uniqueness of PAR when comparing the input used, output produced and processing of the computation employed by the methods of Vogelstein and Cancer5000. The accuracy of the predictions of all three methods is presented in Table 7 using the CGC list as the gold standard. This list contains 153 driver genes that belong to the Signal Transduction pathway. The accuracy of driver gene classification of the Vogelstein and Cancer5000 methods for this pathway were 93.4% and 95.2%, respectively, while that of PAR was a comparable 89.2%. The quality of PAR as a binary classifier was also assessed using the Matthews correlation coefficient (MCC). This measure works well when the two classes are of very different sizes, which is the case for driver gene identification. The driver gene class (1) and the non-driver genes class (0) vary significantly, as the total number of genes being classified far outnumbers the number of known drivers. Combining the sensitivity and specificity resulted in a MCC value of 0.41. This further validates PAR as a valuable driver gene identification method, as in the context of a classification problem it is expected that MCC ranges from 0 (equivalent to a random classifier) to +1 (perfect prediction) with −1 indicating the predictions are always incorrect.

**Table 6:** Process comparison of driver gene prediction methods.

	Base algorithm	Input	Output
PAR	Comparison of pathway and reach similarity to core set of driver genes	Pathways, gene interaction network, driver genes	Candidate driver genes form a pathway
Cancer5000	Mutation frequency, clusters of mutations, location of mutations	Gene mutation, conserved regions of proteins	List of candidate driver genes across the genome
Vogelstein	Frequency and 20% pattern of mutation	Gene mutation	List of candidate driver genes across the genome

**Table 7:** Computational comparison of driver gene prediction methods.

	PAR	Vogelstein	Caner5000
Sensitivity TP/(TP + FN)	0.1176	0.3464	0.4327
Specificity TN/(TN + FP)	0.9770	0.9985	0.9896
Accuracy (TP + TN)/(TP + FP + FN + TN)	89.2%	93.4%	95.2%

Pathway and reach is a novel prediction method that does not use frequency of gene mutations to classify cancer driver genes, which enables it to identify candidate driver genes that are not frequently mutated, oncogenes that are activated by overexpression, and TSGs that are inactivated by suppressed expression. Although the use of mutation recurrence to identify driver genes has had much success, many limitations exist (Lawrence et al., 2013; Waks et al., 2016). PAR provides an alternate and valuable addition to the existing array of methods used to identify candidate driver genes.

## Discussion

We have developed a novel network-based algorithm that employs 2-reach as a metric in conjunction with pathway similarity profiles to identify candidate driver genes using a guilty by resemblance doctrine, which is a unique similarity association approach. The use of a core set of 71 driver genes common to three published lists of cancer driver genes as a reference set is a new approach tested on three separate pathways. There is scope to employ PAR using genes from other pathways and different sets of core driver genes to explore its full potential. No single oncogene or TSG has been implicated in all forms of a particular type of cancer, which makes it difficult to identify oncogenes and TSGs. The deregulation of a pathway may be brought about by alteration of the activity of any one of a number of different genes. PAR avoids the drawbacks of approaches that fail to identify infrequently mutated driver genes, and uses the characteristics of frequently mutated genes as a template.

It has for some time been clear that most cancers can be divided into several different subtypes, with varying prognoses and treatment responses. The guilty by resemblance doctrine, approaches cancer at the pathway and network level, one level above the gene level used by traditional methods. Thus, core pathways that drive the development of cancer are implicated and new driver genes are identified providing potential therapeutic benefits.

The accuracy of PAR is dependent on the quality of gene interaction networks, which can be improved by combining different networks (Ramsahai et al., 2017). Consideration can also be given to the use of pathway analysis tools when choosing the input pathway for PAR. One such tool is APA (Kaushik, Ali & Gupta, 2017) which identifies rewired pathways using patients expression data. Scientists have investigated cancer through the lens of pathway and network analysis, which is showing potential to transform our approach to research into disease etiology and treatment (Creixell et al., 2015). Using m-reach as a metric, protein-protein interaction networks have been transformed into hierarchical structures that enabled genes to be classed at different levels making it possible to compare similarities to driver genes and genes of unknown potential (Mones, Vicsek & Vicsek, 2012). The reach measure has also been shown to be useful in the key player problem (Borgatti, 2003), the solution to which is provided by seeking out the set of nodes, which are maximally connected to other nodes, and the removal of those that cause maximal disruption. One of the solutions to this problem implements the m-reach measure, which is a count of the number of unique nodes reached in m links or less. This provided the motivation to implement the m-reach measure of the gene interaction network in the prediction of driver genes.

The worst case computational complexity of the PAR method is of order O(*n*^2^). The preparation of matrix P and matrix R for all the possible driver genes that could be randomly chosen at the start of the algorithm reduced overall computational time. The performance of the program, therefore, scales well with an increasing number of random selections of the reference driver gene list and the length of this list. We also expect the value of *n* to remain small, as the system has been designed to work with the genes from a particular pathway of interest and a set of reference driver genes. A total of 20 random samples of the 50 driver genes were processed in 5 min on an Intel Core 3.60 GHz processor with 32 GB Ram, while 1,000 random samples of the driver gene list took 45 min on the same machine.

Our top five candidate driver genes *HGF*, *E2F1*, *C6*, *MIF*, and *CDK2* were validated under strict text mining conditions. Limiting our search for these genes to recent papers with the words “cancer” and “patient” together with searching in the title and abstracts only, provided a rigorous condition for assessment. Overexpression of c-met protein combined with an expression of *HGF* is seen in a majority of colorectal cancer cases (Liu, Li & Zhu, 2012). E2F1 has been studied using mouse models of liver cancer to show its role in the cell cycle during cancer progression (Tarangelo et al., 2015), C6 in vivo and in vitro studies has shown its role in apoptosis in multiple human cancer cell lines (Chen et al., 2015). Experimental and clinical studies show that high levels of MIF are found in almost all types of human cancers and are implicated in seemingly all stages of development of tumors (Nobre et al., 2017). CDK2 perturbations in chromosomal stability are known to be pivotal tumorigenic events (Asghar et al., 2015).

## Conclusion

Using PAR we have identified five new candidate cancer driver genes. Our computational results provide insight and offer guidance by shortlisting *HGF*, *E2F1*, *C6*, *MIF*, and *CDK2*. These novel candidate driver genes are potential genes for drug therapy and further research.

Pathway and reach has the capability to be used with any set of pathway genes that may harbor unknown drivers and any set of network genes with known drivers. Both of these features endow PAR with great flexibility and potential for use as a broad investigative tool. This use of pathways and interactions takes into consideration that the development of any cancer is driven by the combined efforts of a number of deregulated pathways brought about by the activation of oncogenes and the deactivation of TSGs. For each deregulated pathway, there are frequently mutated guilty genes that may be used as a reference for the identification of new driver genes.

Our computational results provide insight and offer guidance by shortlisting *HGF*, *E2F1*, *C6*, *MIF*, and *CDK2*. These novel candidate driver genes are potential genes for setting up biological experiments to further validate the reliability of the results.

## Supplemental Information

10.7717/peerj.6979/supp-1 Supplemental Information 1 File S1. PAR results showing the 1000 pvalues using the STRING network on the Signal Transduction network. The gene score and its two components are also included.

10.7717/peerj.6979/supp-2 Supplemental Information 2 File S1.2. The p-value frequency distribution graphs for 50 candidate driver genes.

10.7717/peerj.6979/supp-3 Supplemental Information 3 File S2. Supporting sentences for the novel candidate driver genes.

10.7717/peerj.6979/supp-4 Supplemental Information 4 File S3. R Code with inline results produced using R Markdown, which is fully reproducible.

10.7717/peerj.6979/supp-5 Supplemental Information 5 File S4. R Markdown Code (Rmd file), 3 networks (Rdata file) and other input files (txt and csv files).

10.7717/peerj.6979/supp-6 Supplemental Information 6 Time series of candidate driver gene data.

## Additional Information and Declarations

### Data Availability

The following information was supplied regarding data availability: The R code with inline results are available as Supplemental Files. This was produced using R Markdown, which is fully reproducible. The data used are publicly available and imported from the libraries used.
