# Supplementary material for: Cancer driver genes: a guilty by resemblance doctrine
Source: PeerJ. 2019 Jun 25;7:e6979. doi: 10.7717/peerj.6979 (PMC6598669; doi:10.7717/peerj.6979)
Supplement: Supplemental Information 2 [file peerj-07-6979-s002.pdf]

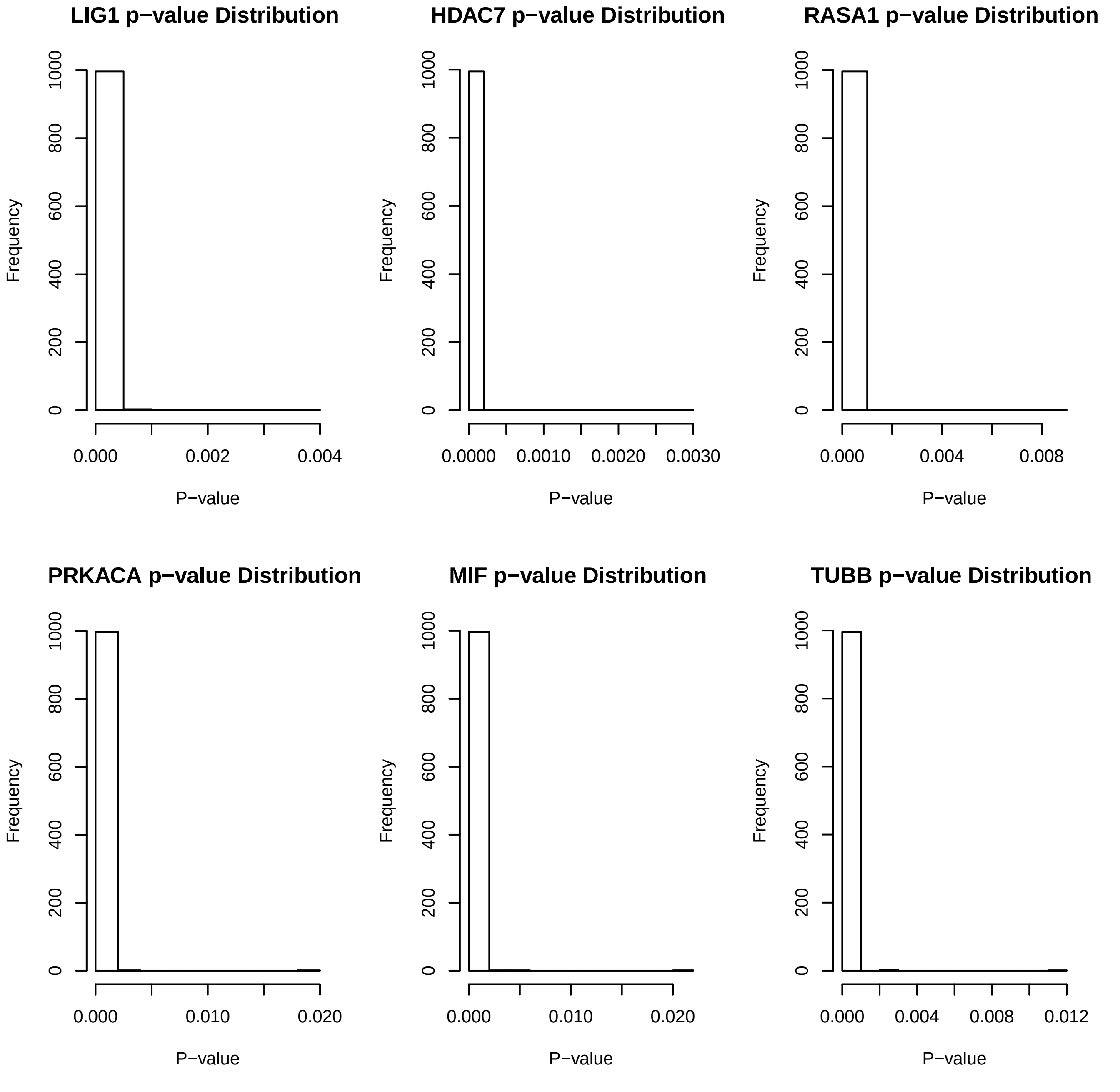
LIG1 p−value Distribution
HDAC7 p−value Distribution
RASA1 p−value Distribution
PRKACA p−value Distribution
MIF p−value Distribution
TUBB p−value Distribution
Frequency
P−value
0
200
400
600
800
1000
0.000
0.002
0.004
0.0000
0.0010
0.0020
0.0030
0.008
0.010
0.020
0.012

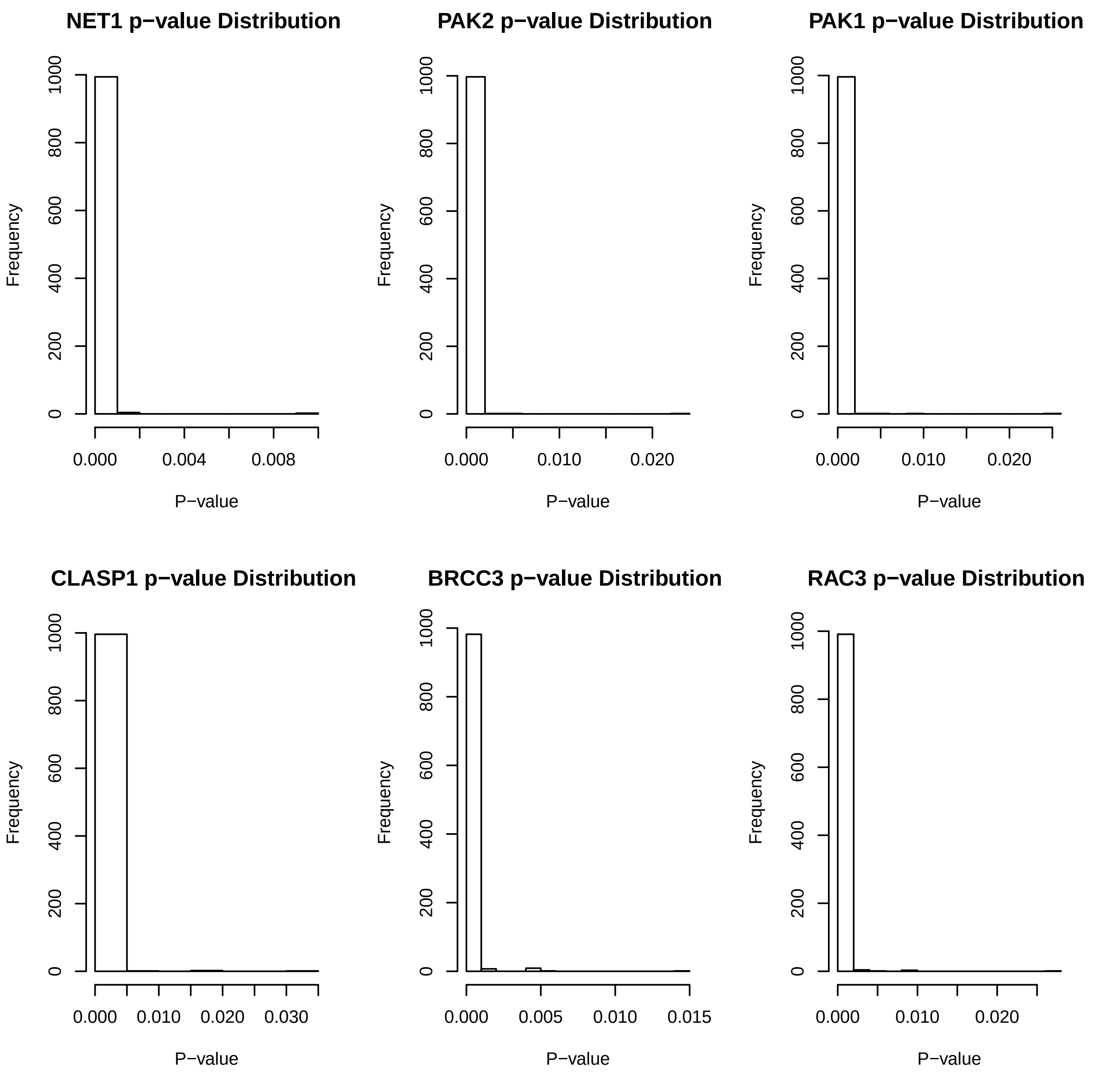
NET1 p−value Distribution
PAK2 p−value Distribution
PAK1 p−value Distribution
CLASP1 p−value Distribution
BRCC3 p−value Distribution
RAC3 p−value Distribution
Frequency
P−value
0
200
400
600
800
1000
0.000
0.004
0.008
0.010
0.020
0.030
0.005
0.015

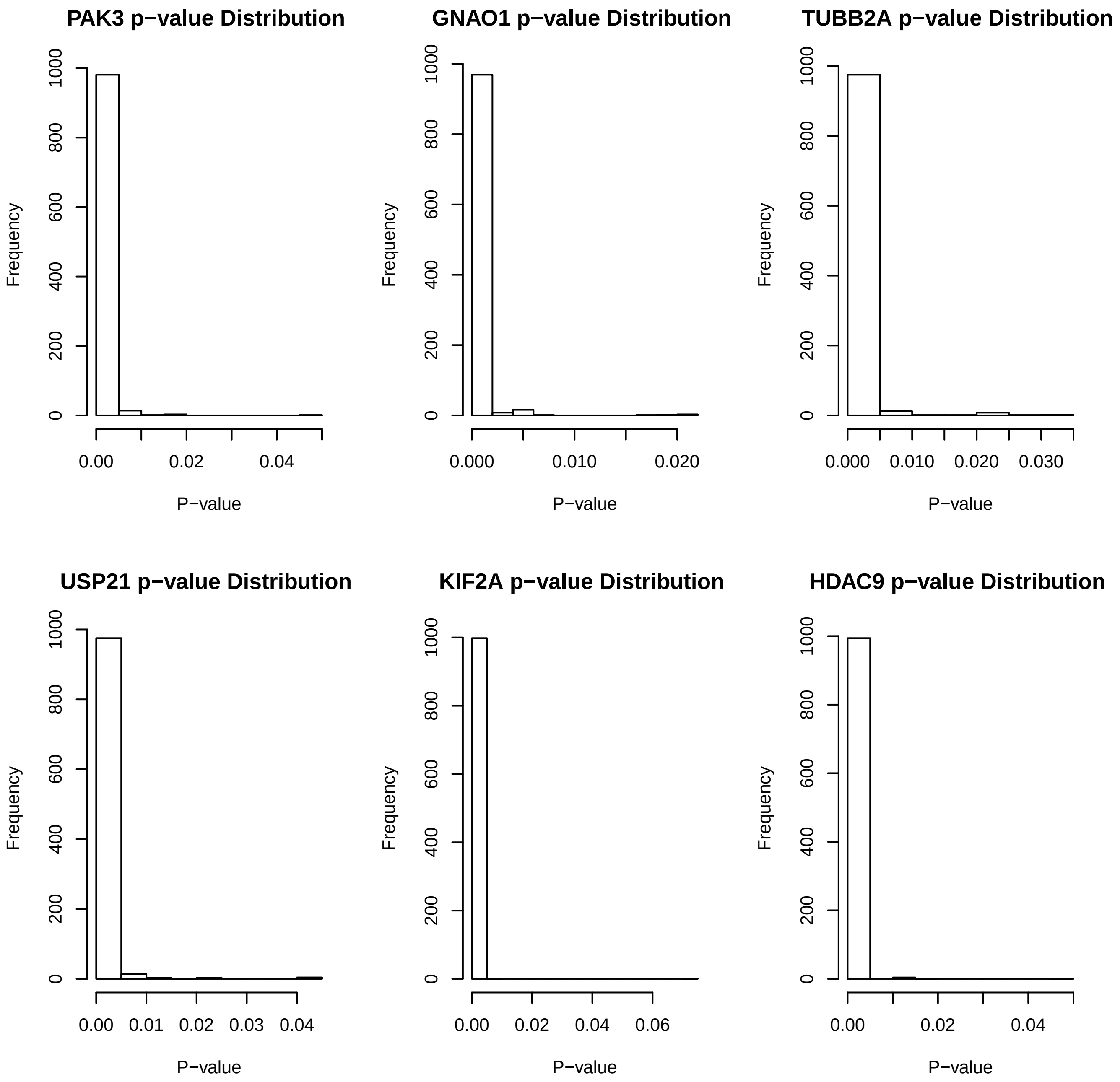
PAK3 p−value Distribution
GNAO1 p−value Distribution
TUBB2A p−value Distribution
USP21 p−value Distribution
KIF2A p−value Distribution
HDAC9 p−value Distribution
Frequency
P−value
0
200
400
600
800
1000
0.00
0.02
0.04
0.000
0.010
0.020
0.030
0.01
0.03
0.06

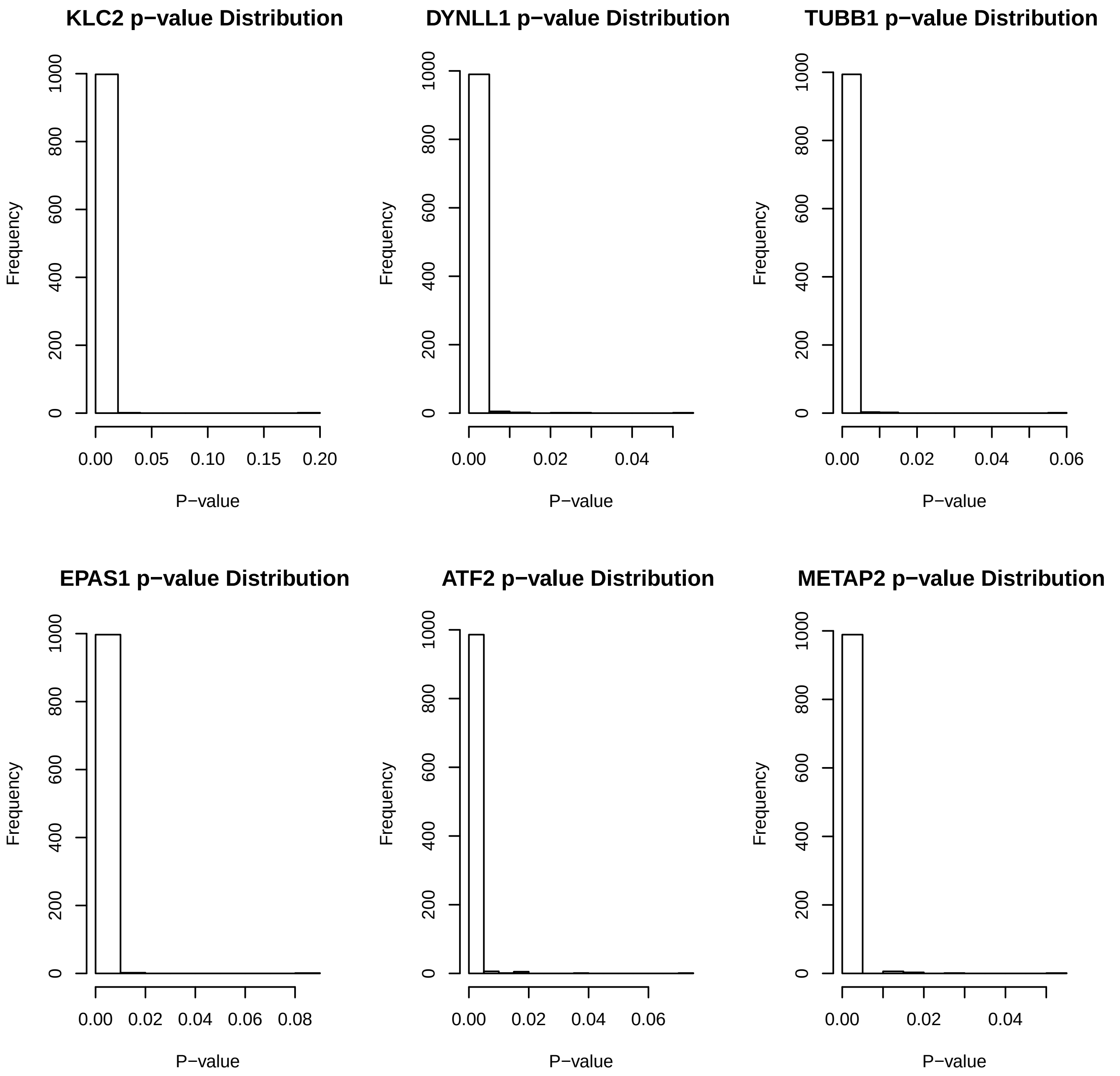

KLC2 p−value Distribution
DYNLL1 p−value Distribution
TUBB1 p−value Distribution
EPAS1 p−value Distribution
ATF2 p−value Distribution
METAP2 p−value Distribution
Frequency
P−value
0
200
400
600
800
1000
0.00
0.05
0.10
0.15
0.20
0.02
0.04
0.06
0.08

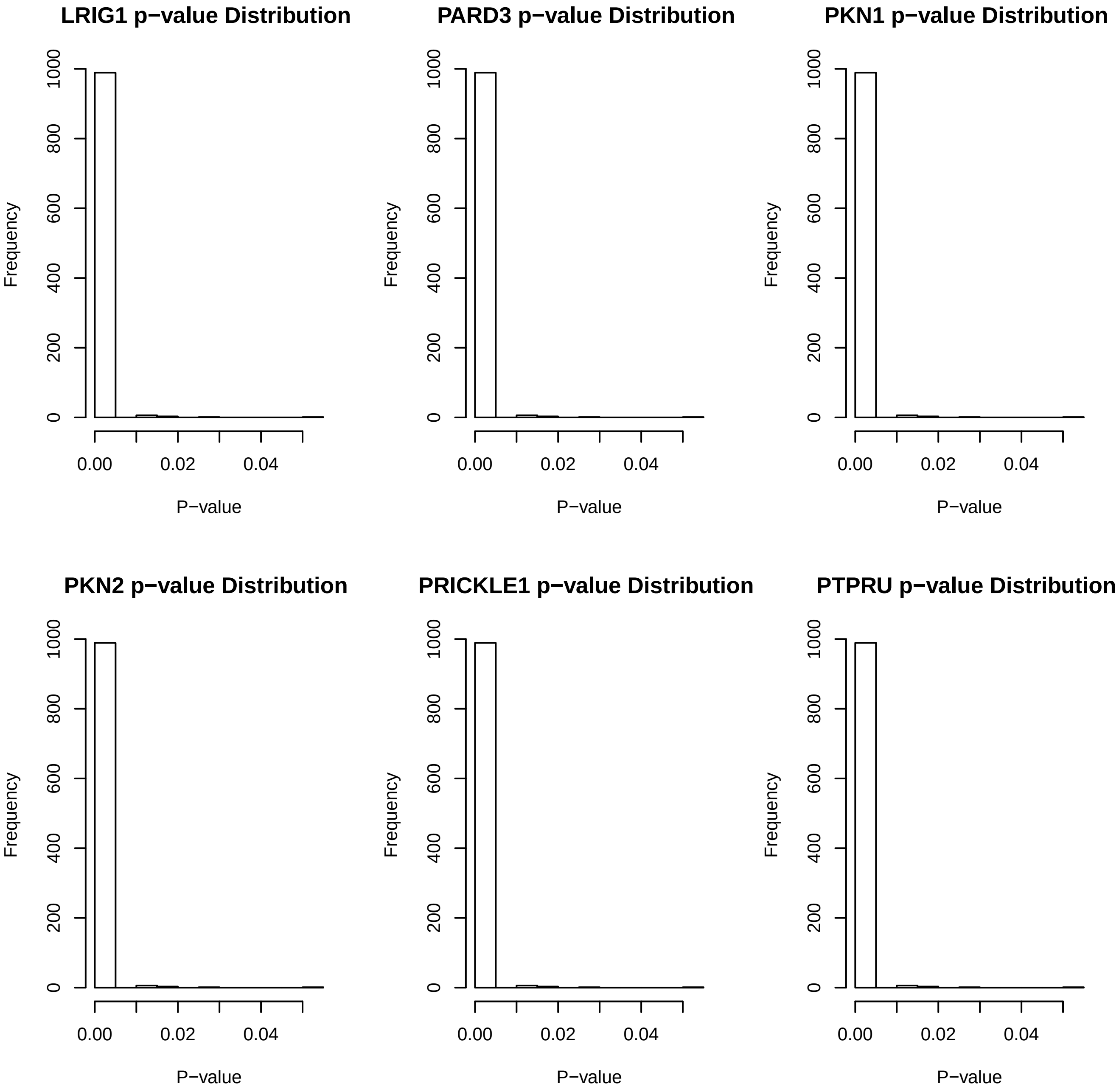
LRIG1 p−value Distribution
PARD3 p−value Distribution
PKN1 p−value Distribution
PKN2 p−value Distribution
PRICKLE1 p−value Distribution
PTPRU p−value Distribution
Frequency
0
200
400
600
800
1000
0.00
0.02
0.04
P−value

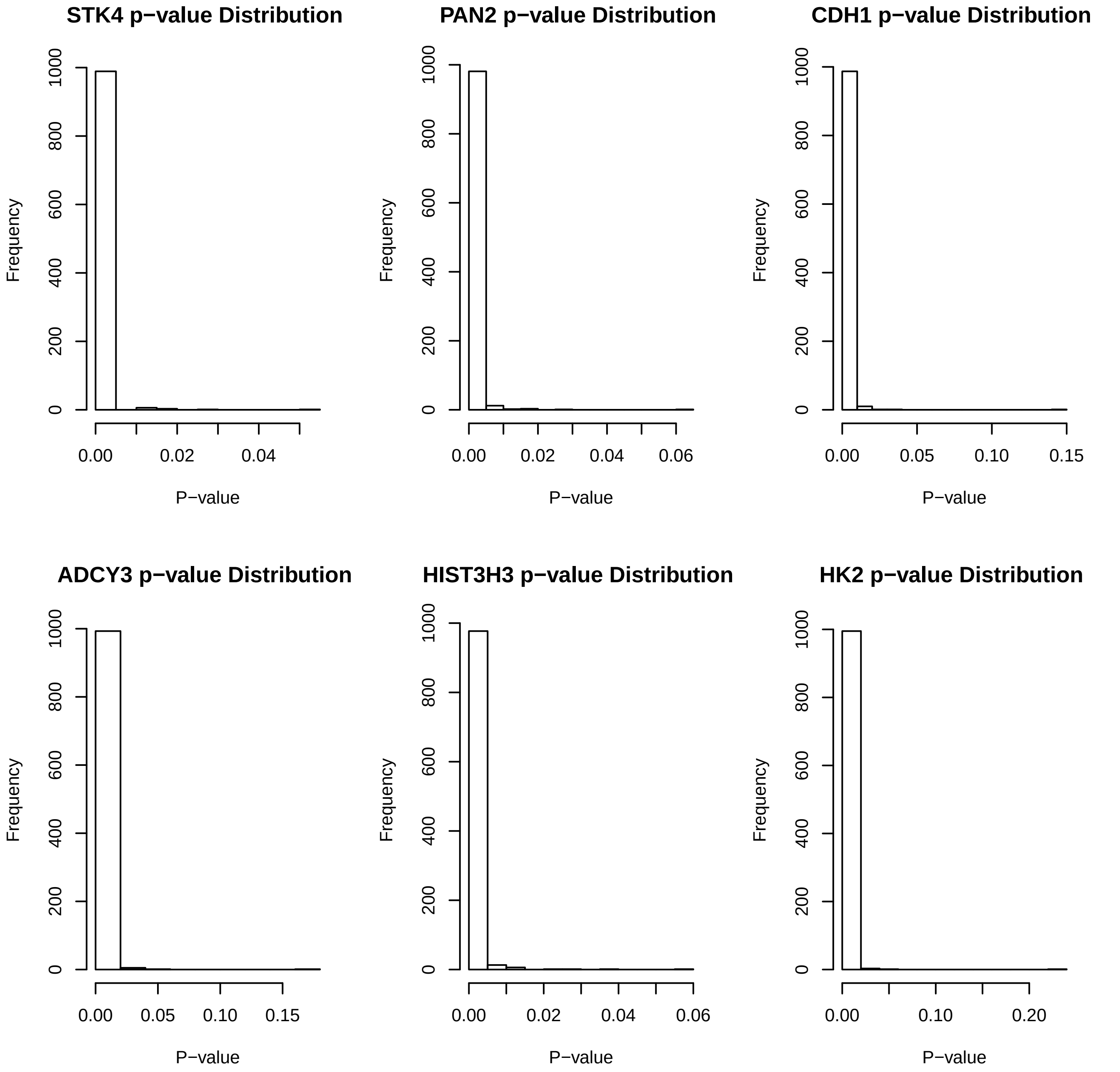
STK4 p−value Distribution
PAN2 p−value Distribution
CDH1 p−value Distribution
ADCY3 p−value Distribution
HIST3H3 p−value Distribution
HK2 p−value Distribution
Frequency
P−value
0
200
400
600
800
1000
0.00
0.02
0.04
0.06
0.05
0.10
0.15
0.20

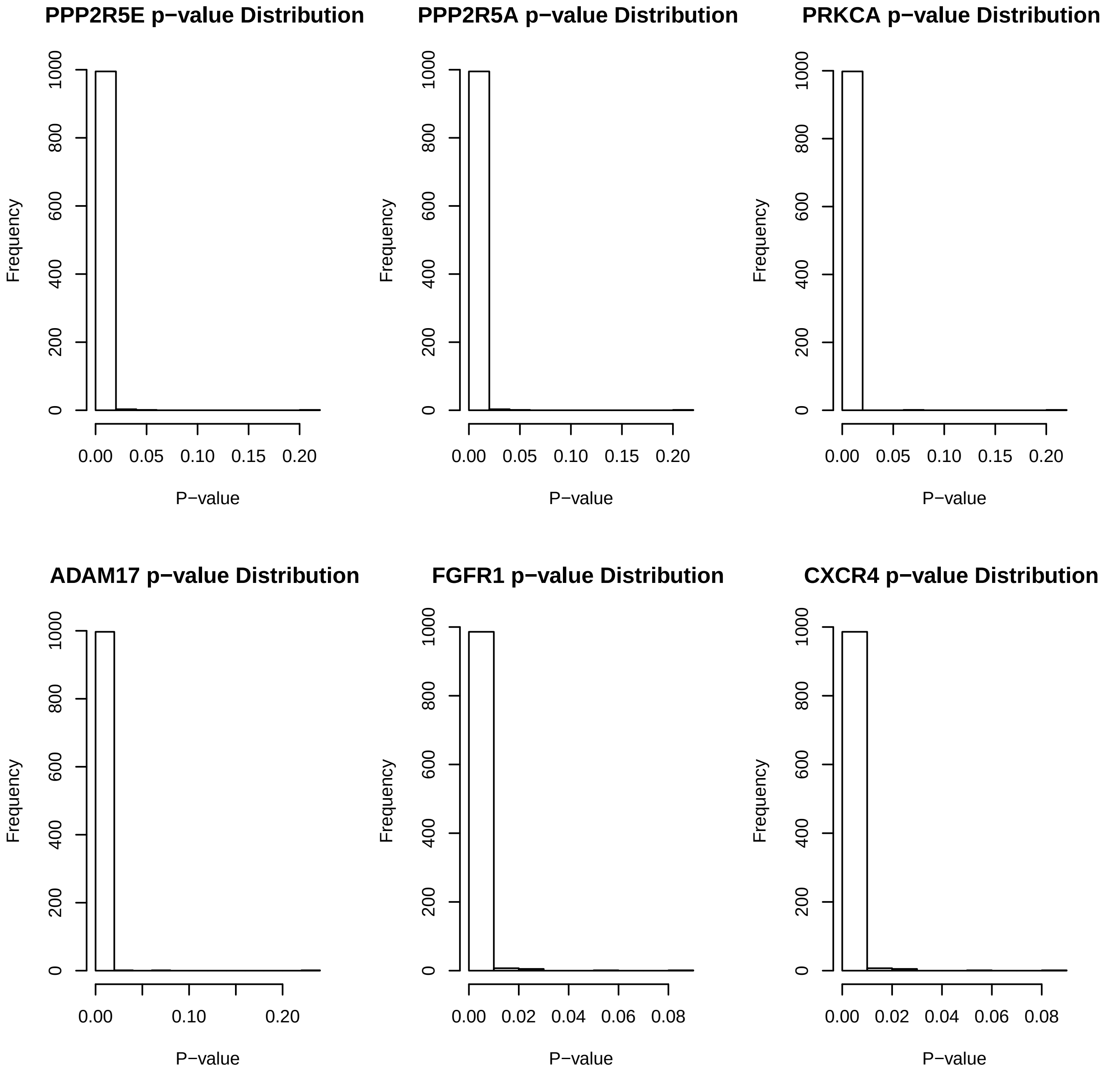
PPP2R5E p−value Distribution
PPP2R5A p−value Distribution
PRKCA p−value Distribution
ADAM17 p−value Distribution
FGFR1 p−value Distribution
CXCR4 p−value Distribution
Frequency
P−value
0
200
400
600
800
1000
0.00
0.05
0.10
0.15
0.20
0.02
0.04
0.06
0.08

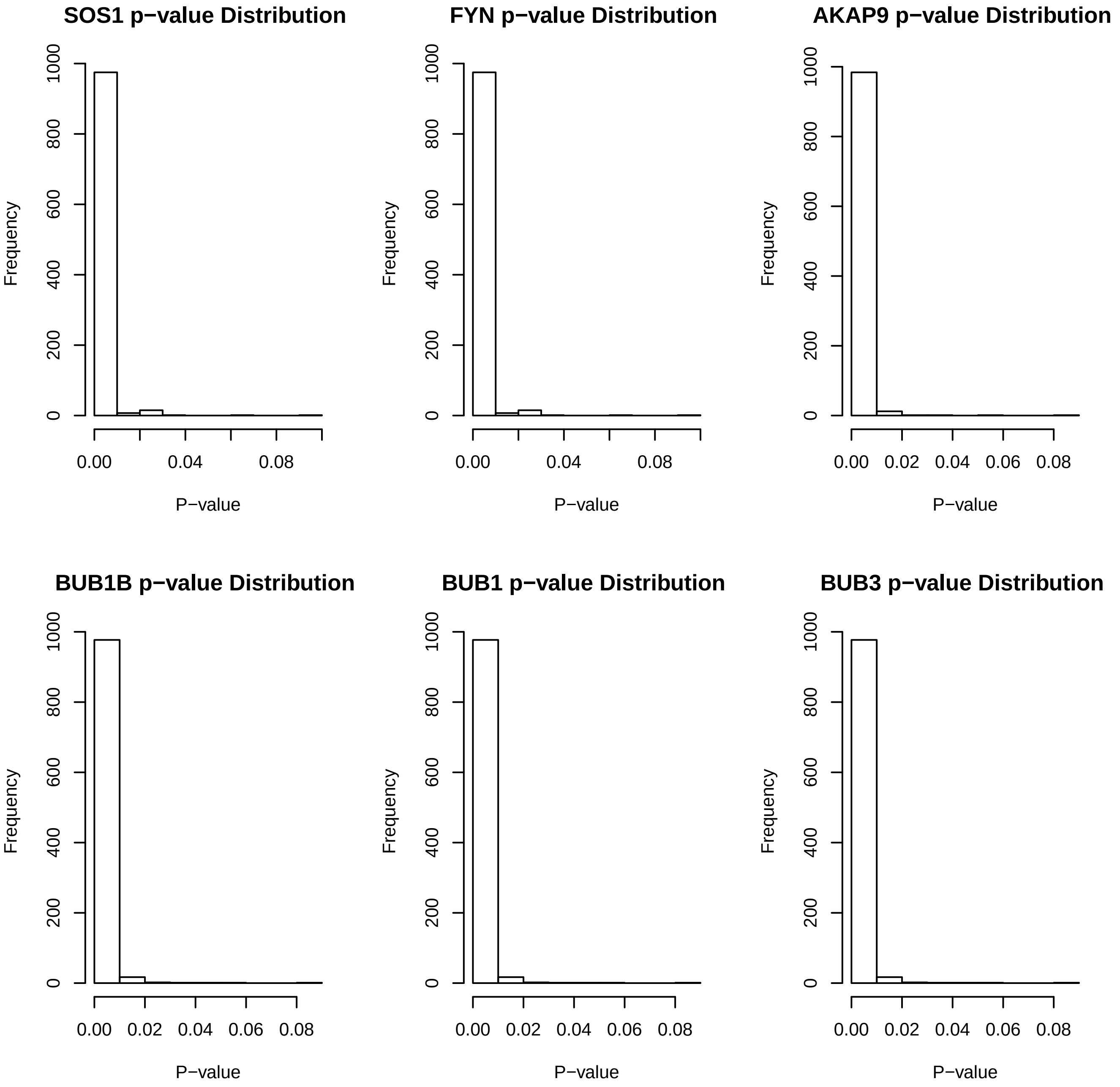
SOS1 p−value Distribution
Frequency
0
200
400
600
800
1000
0.00
0.04
0.08
P−value
FYN p−value Distribution
Frequency
0
200
400
600
800
1000
0.00
0.04
0.08
P−value
AKAP9 p−value Distribution
Frequency
0
200
400
600
800
1000
0.00
0.02
0.04
0.06
0.08
P−value
BUB1B p−value Distribution
Frequency
0
200
400
600
800
1000
0.00
0.02
0.04
0.06
0.08
P−value
BUB1 p−value Distribution
Frequency
0
200
400
600
800
1000
0.00
0.02
0.04
0.06
0.08
P−value
BUB3 p−value Distribution
Frequency
0
200
400
600
800
1000
0.00
0.02
0.04
0.06
0.08
P−value

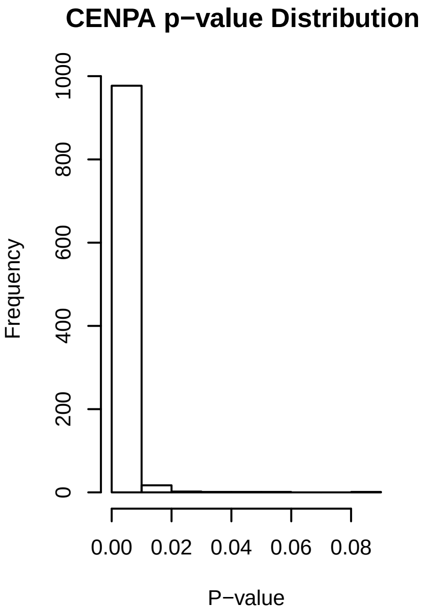
CENPA p−value Distribution
Frequency
0
200
400
600
800
1000
0.00
0.02
0.04
0.06
0.08
P−value

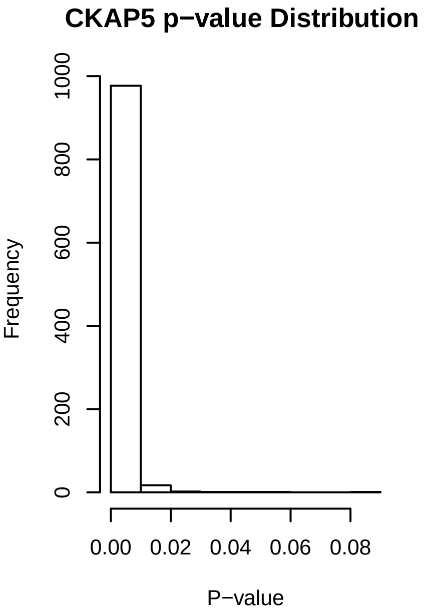
CKAP5 p−value Distribution
Frequency
0
200
400
600
800
1000
0.00
0.02
0.04
0.06
0.08
P−value
