# Supplementary material for: Cancer driver genes: a guilty by resemblance doctrine
Source: PeerJ. 2019 Jun 25;7:e6979. doi: 10.7717/peerj.6979 (PMC6598669; doi:10.7717/peerj.6979)
Supplement: Supplemental Information 4 [file peerj-07-6979-s004.html]

Supplementary code and figures accompanying PAR


# Supplementary code and figures accompanying PAR

Emilie Ramsahai 1, Vrijesh Tripathi 1, Melford John 2

1 Department of Mathematics and Statistics, The University of the West Indies, St Augustine, Trinidad and Tobago 2 Department of Preclinical Sciences, The University of the West Indies, St Augustine, Trinidad and Tobago

This document contains code used in our analysis. It is purely written in R using a series of R and Bioconductor packages. This report has been generated using the knitr R package (http://yihui.name/knitr/). For a complete list of packages and their versions please have a look at the end of this document.

```
#To install the BioConductor libraries use the command
#source("https://bioconductor.org/biocLite.R")
#biocLite("reactome.db")
#biocLite("org.Hs.eg.db")
#The data files are to be placed in your working directory

  rm(list=ls())
  library("reactome.db")
```

```
## Loading required package: AnnotationDbi
```

```
## Loading required package: stats4
```

```
## Loading required package: BiocGenerics
```

```
## Loading required package: parallel
```

```
## 
## Attaching package: 'BiocGenerics'
```

```
## The following objects are masked from 'package:parallel':
## 
##     clusterApply, clusterApplyLB, clusterCall, clusterEvalQ,
##     clusterExport, clusterMap, parApply, parCapply, parLapply,
##     parLapplyLB, parRapply, parSapply, parSapplyLB
```

```
## The following objects are masked from 'package:stats':
## 
##     IQR, mad, sd, var, xtabs
```

```
## The following objects are masked from 'package:base':
## 
##     anyDuplicated, append, as.data.frame, basename, cbind,
##     colMeans, colnames, colSums, dirname, do.call, duplicated,
##     eval, evalq, Filter, Find, get, grep, grepl, intersect,
##     is.unsorted, lapply, lengths, Map, mapply, match, mget, order,
##     paste, pmax, pmax.int, pmin, pmin.int, Position, rank, rbind,
##     Reduce, rowMeans, rownames, rowSums, sapply, setdiff, sort,
##     table, tapply, union, unique, unsplit, which, which.max,
##     which.min
```

```
## Loading required package: Biobase
```

```
## Welcome to Bioconductor
## 
##     Vignettes contain introductory material; view with
##     'browseVignettes()'. To cite Bioconductor, see
##     'citation("Biobase")', and for packages 'citation("pkgname")'.
```

```
## Loading required package: IRanges
```

```
## Loading required package: S4Vectors
```

```
## 
## Attaching package: 'S4Vectors'
```

```
## The following object is masked from 'package:base':
## 
##     expand.grid
```

```
## 
## Attaching package: 'IRanges'
```

```
## The following object is masked from 'package:grDevices':
## 
##     windows
```

```
  library("org.Hs.eg.db")
```

```
##
```

```
  library("igraph")
```

```
## 
## Attaching package: 'igraph'
```

```
## The following object is masked from 'package:IRanges':
## 
##     union
```

```
## The following object is masked from 'package:S4Vectors':
## 
##     union
```

```
## The following objects are masked from 'package:BiocGenerics':
## 
##     normalize, path, union
```

```
## The following objects are masked from 'package:stats':
## 
##     decompose, spectrum
```

```
## The following object is masked from 'package:base':
## 
##     union
```

```
  library(gplots)
```

```
## 
## Attaching package: 'gplots'
```

```
## The following object is masked from 'package:IRanges':
## 
##     space
```

```
## The following object is masked from 'package:S4Vectors':
## 
##     space
```

```
## The following object is masked from 'package:stats':
## 
##     lowess
```

```
  library("sets")
```

```
## 
## Attaching package: 'sets'
```

```
## The following object is masked from 'package:igraph':
## 
##     %>%
```

```
## Analysis can be done with any network
## 3 networks are provided 1.Pathway Commons 2. BioGrid 3. STRING
## The final network is used for processing.
## Ensure the required network is the final network to be loaded
## This arrangement of the code uses the STRING network 

## PATHWAY COMMONS   
  load("pcom.rdata")
  g<-org.Hs.PCommons_DN
  g
```

```
## IGRAPH 3297702 DN-- 15044 249606 -- 
## + attr: name (v/c), geneid (v/n), symbol (v/c), description (v/c),
## | catalysis_precedes (e/n), controls_expression_of (e/n),
## | controls_phosphorylation_of (e/n), controls_state_change_of
## | (e/n), controls_transport_of (e/n)
## + edges from 3297702 (vertex names):
##  [1] PNCK->EIF4G3   PNCK->HSP90AB1 PNCK->HSP90AA1 DPM2->ALG10   
##  [5] DPM2->PIGL     DPM2->ALG10B   AACS->NME1     AACS->NME3    
##  [9] AACS->NME4     AACS->SLC16A7  AACS->CPT1C    AACS->CPT2    
## [13] AACS->PGM2     AACS->OXCT1    AACS->NME2     AACS->PMM2    
## [17] AACS->PPA1     AACS->NME7     AACS->CPT1A    AACS->AOX1    
## + ... omitted several edges
```

```
#BIOGRID-ORGANISM-Homo_sapiens-3.4.163.tab2

  b<-read.csv(file = "BIOGRID-ORGANISM-Homo_sapiens-3.4.163.tab2.txt", sep = "\t")[ ,1:9]
  colnames(b)<-c("V1","V2","V3", "V4", "V5", "V6", "V7", "V8", "V9")
  b1<-b[,8:9]
  bg <- graph.data.frame(b1, directed=FALSE)
  g<-bg

## STRING
  sppi<-read.table('1_ppi_string.txt',header = FALSE, sep='\t')
  sg <- graph.data.frame(sppi, directed=FALSE)
  g<-sg
  
## ALL Reactome pathways

  pathways <- toTable(reactomePATHNAME2ID)
  pathwaysSelectedSpecies <- pathways[grep("Homo sapiens: ", iconv(pathways$path_name)), ]
  head(pathwaysSelectedSpecies[,2] )
```

```
## [1] "Homo sapiens: 2-LTR circle formation"                                         
## [2] "Homo sapiens: 5-Phosphoribose 1-diphosphate biosynthesis"                     
## [3] "Homo sapiens: A tetrasaccharide linker sequence is required for GAG synthesis"
## [4] "Homo sapiens: ABC transporter disorders"                                      
## [5] "Homo sapiens: ABC transporters in lipid homeostasis"                          
## [6] "Homo sapiens: ABC-family proteins mediated transport"
```

```
#Input reference List of driver genes, and Pathway of interest (Signal transduction)
vogel<-read.table('vogel.txt',header = FALSE, sep='\t')
c5000<-read.table('Cancer5000.csv',header = FALSE, sep='\t')
census<-read.table('census.csv',header = FALSE, sep=',')
ven1<-venn( list(Cancer5000=c5000$V1,Vogelstein=vogel$V1,CGC=census$V1))
```

```
isect <- attr(ven1, "intersection")
refDriver<-isect$`Cancer5000:Vogelstein:CGC`

dfrefDriver<-data.frame(refDriver)

PidSymbol<-dfrefDriver$refDriver

indx<-V(g)$name %in% PidSymbol
subv<- V(g)[indx]
net2 <- induced.subgraph(graph=g,vids=subv)
dg <- decompose.graph(net2) 
DriverNetwork<-dg[[1]]  
DriverNetwork
```

```
## IGRAPH d9e81d6 UN-- 68 643 -- 
## + attr: name (v/c), V3 (e/n)
## + edges from d9e81d6 (vertex names):
##  [1] AKT1  --ASXL1  AKT1  --BRAF   AKT1  --BRCA1  ATM   --BRCA1 
##  [5] CASP8 --CDH1   AKT1  --CDKN2A ATM   --CDKN2A BRCA1 --CDKN2A
##  [9] AKT1  --CREBBP APC   --CREBBP ARID1A--CREBBP ATM   --CREBBP
## [13] BRCA1 --CREBBP CDH1  --CREBBP AKT1  --CTNNB1 APC   --CTNNB1
## [17] BRCA1 --CTNNB1 CASP8 --CTNNB1 CDH1  --CTNNB1 CREBBP--CTNNB1
## [21] AKT1  --EGFR   BRAF  --EGFR   CDH1  --EGFR   CDKN2A--EGFR  
## [25] CTNNB1--EGFR   AKT1  --EP300  ARID1A--EP300  ATM   --EP300 
## [29] BRCA1 --EP300  CDH1  --EP300  CDKN2A--EP300  CREBBP--EP300 
## + ... omitted several edges
```

```
plot(DriverNetwork,vertex.label=NA)
```

```
VdfDriverNetwork<-data.frame(V1 = V(DriverNetwork)$name)

## Analysis can be done with any pathway
## 3 pathways were tested 1.Gene Expression 2. Immune System 3. Signal Transduction
## The final pathway is used for processing.
## Ensure the required pathway is the final pathway to be loaded
## This arrangement of the code uses the Signal Transduction network 

pathwayOfInterest ="Homo sapiens: Gene expression (Transcription)"
pathwayOfInterest ="Homo sapiens: Immune System"
pathwayOfInterest = "Homo sapiens: Signal Transduction"


xx <- as.list(reactomePATHNAME2ID) 
pathwayOfInterestID = as.character(xx[pathwayOfInterest])
xx2 <- as.list(reactomePATHID2EXTID) 
PidGene<-(xx2[pathwayOfInterestID])
PidGeneid<-PidGene[[1]]
PathwayGeneList<-as.character(PidGeneid)
PathwayGeneListAlias<-data.frame(select(org.Hs.eg.db, PathwayGeneList, c("ALIAS"), "ENTREZID"))
```

```
## 'select()' returned many:many mapping between keys and columns
```

```
PidSymbol<-PathwayGeneListAlias$ALIAS

indx<-V(g)$name %in% PidSymbol
subv<- V(g)[indx]
net2 <- induced.subgraph(graph=g,vids=subv)

#decompose to determine final ST connected pathway

dg <- decompose.graph(net2) 
PathwayNetwork<-dg[[1]] 
PathwayNetwork
```

```
## IGRAPH daac892 UN-- 2655 207992 -- 
## + attr: name (v/c), V3 (e/n)
## + edges from daac892 (vertex names):
##  [1] AKT1    --AR       AKT1    --ARHGEF12 AKT1    --ARHGEF7 
##  [4] ARHGEF12--ARHGEF7  ARPC2   --ARPC4    APC     --ASH2L   
##  [7] AR      --ASH2L    ARPC2   --ATP6V1B1 ATP6V1B1--ATP6V1D 
## [10] AKT1    --AURKB    ASH2L   --AURKB    APC     --AXIN1   
## [13] AKT1    --BAD      AKT1    --BCL2     BAD     --BCL2    
## [16] AR      --BIRC2    AURKB   --BIRC2    BCL2    --BIRC2   
## [19] AR      --BIRC3    AURKB   --BIRC3    BCL2    --BIRC3   
## [22] BIRC2   --BIRC3    APC     --BIRC5    AR      --BIRC5   
## + ... omitted several edges
```

```
plot(PathwayNetwork,vertex.label=NA)
```

```
VdfPathwayNetwork<-data.frame(V1 = V(PathwayNetwork)$name)
```

```
Vdf<-data.frame(V1 = V(g)$name)

x <- org.Hs.egSYMBOL2EG 
mapped_genes <- mappedkeys(x) 
VdfPathwayNetwork2<-as.character(VdfPathwayNetwork$V1)
indx<-(mapped_genes %in% VdfPathwayNetwork2)
VdfPathwayNetwork1<-mapped_genes[indx]
VdfPathwayNetworkEZ<-data.frame(select(org.Hs.eg.db, VdfPathwayNetwork1, c("ENTREZID","GENENAME"), "ALIAS"))
```

```
## 'select()' returned 1:many mapping between keys and columns
```

```
dim(VdfPathwayNetworkEZ)
```

```
## [1] 2877    3
```

```
head(VdfPathwayNetworkEZ)
```

```
##   ALIAS ENTREZID                                           GENENAME
## 1   A2M        2                              alpha-2-macroglobulin
## 2 ABCA4       24          ATP binding cassette subfamily A member 4
## 3  ABL1       25 ABL proto-oncogene 1, non-receptor tyrosine kinase
## 4   ABR       29          ABR, RhoGEF and GTPase activating protein
## 5  ACTB       60                                         actin beta
## 6  ACTB   728378                POTE ankyrin domain family member F
```

```
VdfDriverNetworkc<-as.character(VdfDriverNetwork$V1)
VdfDriverNetworkEZ<-data.frame(select(org.Hs.eg.db, VdfDriverNetworkc, c("ENTREZID","GENENAME"), "ALIAS"))
```

```
## 'select()' returned 1:many mapping between keys and columns
```

```
dim(VdfDriverNetworkEZ)
```

```
## [1] 74  3
```

```
head(VdfDriverNetworkEZ)
```

```
##    ALIAS ENTREZID
## 1   AKT1      207
## 2    APC      324
## 3    APC     5624
## 4 ARID1A     8289
## 5  ASXL1   171023
## 6    ATM      472
##                                                     GENENAME
## 1                              AKT serine/threonine kinase 1
## 2                       APC, WNT signaling pathway regulator
## 3 protein C, inactivator of coagulation factors Va and VIIIa
## 4                              AT-rich interaction domain 1A
## 5     additional sex combs like 1, transcriptional regulator
## 6                                ATM serine/threonine kinase
```

```
size<-nrow(VdfPathwayNetworkEZ)     
size1<-nrow(VdfDriverNetworkEZ)
Jindex<-matrix(0,size,size1)        

colnames(Jindex)<-VdfDriverNetworkEZ$ENTREZID
rownames(Jindex)<-VdfPathwayNetworkEZ$ENTREZID

xx1 <- as.list(reactomeEXTID2PATHID)
for (j in 1:size){

nvgene<-VdfPathwayNetworkEZ$ENTREZID[j]

for(i in 1:size1) {
valid<-FALSE
valid1<-FALSE
valid2<-FALSE


# Calculate jcard index for 
vgene<-VdfDriverNetworkEZ$ENTREZID[i]

EZ2Pid<-(xx1[vgene])
if (!(is.null(EZ2Pid[[1]]))){
valid1<-TRUE
dfvEZ2Pid<-stack(EZ2Pid)
}


EZ2Pid<-(xx1[nvgene])
if (!(is.null(EZ2Pid[[1]]))){
    valid2<-TRUE
    dfnvEZ2Pid<-stack(EZ2Pid)
}

# Similarity between pathways 


if (valid1 && valid2){
c<-dfnvEZ2Pid$values
a<-as.character(c)
A<-as.set(a)

c<-dfvEZ2Pid$values
B<-as.set(c)
Jindex[j,i]<-set_similarity(A, B)


} # if valid1
} # for i
} #for j

dim(Jindex)
```

```
## [1] 2877   74
```

```
Jindex[1:10,1:16]
```

```
##               207        324       5624       8289     171023        472
## 2      0.02970297 0.02222222 0.11538462 0.00000000 0.00000000 0.00000000
## 24     0.05208333 0.07500000 0.00000000 0.00000000 0.00000000 0.00000000
## 25     0.09649123 0.01515152 0.02040816 0.10000000 0.00000000 0.14666667
## 29     0.03125000 0.02500000 0.00000000 0.00000000 0.00000000 0.00000000
## 60     0.09027778 0.06451613 0.03797468 0.04109589 0.05882353 0.01724138
## 728378 0.00000000 0.00000000 0.00000000 0.00000000 0.00000000 0.00000000
## 71     0.08870968 0.04054054 0.00000000 0.00000000 0.00000000 0.00000000
## 88     0.02803738 0.01960784 0.02941176 0.00000000 0.00000000 0.00000000
## 91     0.02150538 0.02777778 0.00000000 0.00000000 0.00000000 0.00000000
## 92     0.02127660 0.02702703 0.00000000 0.00000000 0.00000000 0.00000000
##               673        672        841        999      51343      1029
## 2      0.01851852 0.00000000 0.01923077 0.11764706 0.00000000 0.0000000
## 24     0.06122449 0.00000000 0.02040816 0.06060606 0.00000000 0.0000000
## 25     0.01333333 0.17460317 0.04225352 0.07272727 0.05263158 0.1355932
## 29     0.02040816 0.00000000 0.04347826 0.06451613 0.00000000 0.0000000
## 60     0.14893617 0.04950495 0.02912621 0.12345679 0.01098901 0.0312500
## 728378 0.00000000 0.00000000 0.00000000 0.00000000 0.00000000 0.0000000
## 71     0.19444444 0.00000000 0.03703704 0.16949153 0.01449275 0.0000000
## 88     0.19607843 0.00000000 0.01724138 0.04761905 0.00000000 0.0000000
## 91     0.02222222 0.00000000 0.02325581 0.03571429 0.00000000 0.0000000
## 92     0.02173913 0.00000000 0.02272727 0.03448276 0.00000000 0.0000000
##              1387       1499       1956       2033
## 2      0.02325581 0.02941176 0.01369863 0.01904762
## 24     0.03658537 0.04687500 0.07575758 0.02970297
## 25     0.10101010 0.13750000 0.06741573 0.11304348
## 29     0.01219512 0.03174603 0.04545455 0.00990099
## 60     0.09302326 0.17142857 0.12389381 0.14285714
## 728378 0.00000000 0.00000000 0.00000000 0.00000000
## 71     0.05309735 0.18823529 0.14130435 0.04545455
## 88     0.02173913 0.02702703 0.05263158 0.01801802
## 91     0.02597403 0.03389831 0.03174603 0.02083333
## 92     0.02564103 0.03333333 0.03125000 0.02061856
```

```
Jindex[Jindex > 0] <- 1
image( Jindex, xaxt= "n", yaxt= "n" )
```

```
reach2=function(x){         
r=vector(length=vcount(x))
for (i in 1:vcount(x)){
n=neighborhood(x,2,nodes=i)
ni=unlist(n)
l=length(ni)
r[i]=(l)/vcount(x)}
r}

r2<-reach2(g)
Vdf$r2<-r2
range<-quantile(r2)

size<-length(VdfPathwayNetwork2)
size1<-length(VdfDriverNetworkc)
RLevel<-matrix(0,size,size1)        

colnames(RLevel)<-VdfDriverNetworkc
rownames(RLevel)<-VdfPathwayNetwork2

for (j in 1:size){
nvgene<-VdfPathwayNetwork2[j]
  
for(i in 1:size1) {

vgene<-VdfDriverNetworkc[i]
  
geneid1<-as.character(vgene)
geneid2<-as.character(nvgene)
r21<-Vdf[Vdf$V1==geneid1,]$r2
r22<-Vdf[Vdf$V1==geneid2,]$r2
r21I<-findInterval(r21,range)
r22I<-findInterval(r22,range)

if (r21I==r22I)
    RLevel[j,i]<-1


} # for i
} #for j

RLevel[1:10,1:16]
```

```
##          AKT1 APC ARID1A ASXL1 ATM BRAF BRCA1 CASP8 CDH1 CDKN2A CREBBP
## AKT1        1   1      1     0   1    1     1     1    1      1      1
## AMOT        0   0      0     1   0    0     0     0    0      0      0
## APC         1   1      1     0   1    1     1     1    1      1      1
## APOA1       0   0      0     0   0    0     0     0    0      0      0
## AR          1   1      1     0   1    1     1     1    1      1      1
## ARHGEF12    1   1      1     0   1    1     1     1    1      1      1
## ARHGEF7     1   1      1     0   1    1     1     1    1      1      1
## ARPC2       1   1      1     0   1    1     1     1    1      1      1
## ARPC4       0   0      0     1   0    0     0     0    0      0      0
## ASH2L       1   1      1     0   1    1     1     1    1      1      1
##          CTNNB1 EGFR EP300 ERBB2 FBXW7
## AKT1          1    1     1     1     1
## AMOT          0    0     0     0     0
## APC           1    1     1     1     1
## APOA1         0    0     0     0     0
## AR            1    1     1     1     1
## ARHGEF12      1    1     1     1     1
## ARHGEF7       1    1     1     1     1
## ARPC2         1    1     1     1     1
## ARPC4         0    0     0     0     0
## ASH2L         1    1     1     1     1
```

```
#######################

df1Jindex<-as.data.frame(as.table(Jindex))
df2Jindex<-merge(df1Jindex,VdfDriverNetworkEZ,by.x="Var2",by.y="ENTREZID")
df3Jindex<-merge(df2Jindex,VdfPathwayNetworkEZ,by.x="Var1",by.y="ENTREZID")
df4Jindex<-df3Jindex[,c("Freq","ALIAS.x","ALIAS.y")]
PMatrix3<-df4Jindex
ssize<-50

#1000 repetitions

for (j in 1:1000) {

dim(PMatrix3)   
head(PMatrix3)

df2<-PMatrix3
colnames(df2) <- c("PFreq","Var2","Var1")
head(df2)
drivers<-data.frame(unique(df2$Var2))

smpl <- drivers[sample(nrow(drivers), ssize),]
indx<-df2$Var2 %in% smpl
dfPMatrix3<-df2[indx,]
df2<-dfPMatrix3
head(df2)
tail(df2)


dim(PMatrix3)   
dim(dfPMatrix3)
dim(df2)
dim(RLevel) 

df1Jindex<-as.data.frame(as.table(RLevel))
df3<-df1Jindex
dim(df3) 


indx<-df3$Var2 %in% smpl
dfJindex<-df3[indx,]
df3<-dfJindex
head(df3)
tail(df3)

dim(Jindex)
dim(dfJindex) 
dim(df3)

################################################

colnames(df2) <- c("PFreq","Var2","Var1")
d4<-merge(df2, df3, by = c("Var1","Var2"), all.x = TRUE)
d4[is.na(d4)] <- 0
colnames(d4)[1] <- "cgene"
colnames(d4)[2] <- "dgene"
colnames(d4)[3] <- "Pscore"
colnames(d4)[4] <- "Nscore"
head(d4)

dftbl<-data.frame(table(d4$Nscore,d4$Pscore,d4$cgene))
head(dftbl)
dim(dftbl)
colnames(dftbl)[1]<-"gene"
colnames(dftbl)[2]<-"path"
colnames(dftbl)[3]<-"cgene"
head(dftbl)

df2<-dftbl
head(df2)
candidate<- data.frame(table(df2$cgene))
ngene<-nrow(candidate)
tab <- t(matrix(nrow=2,ncol=2))

for (i in 1:ngene) {
    t<-(i-1)*4
    tab[1,1]<-df2[t+1,4]
    tab[2,1]<-df2[t+2,4]
    tab[1,2]<-df2[t+3,4]
    tab[2,2]<-df2[t+4,4]
    f<-fisher.test(tab)
    candidate[i,"Freq"]<-f$p.value
}


if (j==1){
  gene1<-candidate
  candidate$Var1     <- NULL
  colnames(candidate)<-paste('Random', j, sep='')
  gene<-candidate
}  
if (j>1){
 
candidate$Var1     <- NULL
colnames(candidate)<-paste('Random', j, sep='')
gene<-cbind(gene,candidate)
gene1<-cbind(gene1,candidate) 
}


}

candidate<- data.frame(table(df2$cgene))
rownames(gene)<-candidate$Var1

##p-values adjusted to control the False Discovery Rate (FDR) 
##using the Benjamini-Hochberg procedure for multiple-testing correction. 

ngene<-nrow(gene)
for (i in 1:ngene) {
adjPval<-gene[i,1:1000]
gene[i,1:1000]<-round(p.adjust(adjPval, "BH"), 3)
}

x<-as.matrix(gene)
heatmap.2(x, col=redgreen(75),  dendrogram= "row", trace="none",density.info="none",labRow=" ", labCol=" ")
```

```
 sessionInfo()
```

```
## R version 3.5.1 (2018-07-02)
## Platform: x86_64-w64-mingw32/x64 (64-bit)
## Running under: Windows 10 x64 (build 17134)
## 
## Matrix products: default
## 
## locale:
## [1] LC_COLLATE=English_United States.1252 
## [2] LC_CTYPE=English_United States.1252   
## [3] LC_MONETARY=English_United States.1252
## [4] LC_NUMERIC=C                          
## [5] LC_TIME=English_United States.1252    
## 
## attached base packages:
## [1] parallel  stats4    stats     graphics  grDevices utils     datasets 
## [8] methods   base     
## 
## other attached packages:
##  [1] sets_1.0-18          gplots_3.0.1         igraph_1.2.2        
##  [4] org.Hs.eg.db_3.6.0   reactome.db_1.64.0   AnnotationDbi_1.42.1
##  [7] IRanges_2.14.10      S4Vectors_0.18.3     Biobase_2.40.0      
## [10] BiocGenerics_0.26.0 
## 
## loaded via a namespace (and not attached):
##  [1] Rcpp_0.12.18       knitr_1.20         magrittr_1.5      
##  [4] bit_1.1-14         stringr_1.3.1      blob_1.1.1        
##  [7] caTools_1.17.1.1   tools_3.5.1        KernSmooth_2.23-15
## [10] DBI_1.0.0          gtools_3.8.1       htmltools_0.3.6   
## [13] yaml_2.2.0         bit64_0.9-7        rprojroot_1.3-2   
## [16] digest_0.6.15      bitops_1.0-6       memoise_1.1.0     
## [19] evaluate_0.12      RSQLite_2.1.1      rmarkdown_1.10    
## [22] gdata_2.18.0       stringi_1.2.4      compiler_3.5.1    
## [25] backports_1.1.2    pkgconfig_2.0.1
```

```
#devtools::session_info()
```
