# Supplementary material for: Cancer driver genes: a guilty by resemblance doctrine
Source: PeerJ. 2019 Jun 25;7:e6979. doi: 10.7717/peerj.6979 (PMC6598669; doi:10.7717/peerj.6979)
Supplement: Supplemental Information 6 [file peerj-07-6979-s006.docx]

**Time series of candidate driver gene data**

| **Gene** | **2018**  **PMID** | **2017**  **PMID** | **2016**  **PMID** | **Gene** | **2018**  **PMID** | **2017**  **PMID** | **2016**  **PMID** |
| --- | --- | --- | --- | --- | --- | --- | --- |
| HGF | *30059715*  *29939324*  *29785126*  *29435981*  *29371783* | 29209570  29050231  28938541  28836864  28831645  28751311  28673936  28644771  28595915  28545495  28416482  28341789  28286925  28280216  28096505  25068470 | 27843623  27016342  27706656  27528224  27139908  27087375  26934743  26750997  26676563  26667487  26503729  26219898 | SRC | 29940643  29937990  29739791  29726962  29553850  29435137  29315500  29190494 | 29435137  29212027  29192740  29190494  28272410  28049763  29511478 | 27861374  27835901  27756880  27439479  27438149  27418135  26936507  26848862  26676753  25618371  25444439 |
| E2F1 | *30036377*  *29950445*  *29633513*  *29596435*  *29547938*  *29448242*  *29217453* | 29217453  29108454  28944330  28753861  28716024  28104681  28074012  28068326 | 27223087  26959119  26856934  29034103  26623722  26575952  26475334  26350215  26349752 | MIF | 29864117 | 29059216  28769740  28542626  28218903  27721403 | 27721403  27197190  27145382  26981417  26980748  26941846  26783288  26719579 |
| CDK2 | *29360538* | 28753861  28595260  27983933 | 27983933  27569395  27337954  27486754  27020857  26935528  26890070 | C6 | 29540297  29369202  28887680 | 28887680 | 28053744  27680947  27379390  27198662  27081081 |
